# Genetic gains in early maturing maize hybrids developed by the International Maize and Wheat Improvement Center in Southern Africa during 2000–2018

**DOI:** 10.3389/fpls.2023.1321308

**Published:** 2024-01-16

**Authors:** Amsal Tarekegne, Dagne Wegary, Jill E. Cairns, Mainassara Zaman-Allah, Yoseph Beyene, Demewoz Negera, Adefris Teklewold, Kindie Tesfaye, MacDonald B. Jumbo, Biswanath Das, Egas J. Nhamucho, Kelvin Simpasa, Kesbell K. E. Kaonga, Kingstone Mashingaidze, Ndhlela Thokozile, Xavier Mhike, Boddupalli M. Prasanna

**Affiliations:** ^1^ Global Maize Program, International Maize and Wheat Improvement Centre (CIMMYT), Harare, Zimbabwe; ^2^ Global Maize Program, International Maize and Wheat Improvement Centre (CIMMYT), Nairobi, Kenya; ^3^ Global Maize Program, International Maize and Wheat Improvement Centre (CIMMYT), Addis Ababa, Ethiopia; ^4^ Sustianable Agrifood Systems Program, International Maize and Wheat Improvement Centre (CIMMYT), Addis Ababa, Ethiopia; ^5^ Crop Improvement Program, International Crops Research Institute for Semi-Arid Tropics, Bamako, Mali; ^6^ Instituto de Investigação Agrária de Moçambique (IIAM), Chokwe, Mozambique; ^7^ Seed Co Zambia International, Lusaka, Zambia; ^8^ Chitedze Research Station, Lilongwe, Malawi; ^9^ Agricultural Research Council-Grain Crop Institute, Potchefstroom, South Africa

**Keywords:** drought tolerance, early-maturity, genetic gains, hybrids, stress tolerance

## Abstract

Genetic gain estimation in a breeding program provides an opportunity to monitor breeding efficiency and genetic progress over a specific period. The present study was conducted to (i) assess the genetic gains in grain yield of the early maturing maize hybrids developed by the International Maize and Wheat Improvement Center (CIMMYT) Southern African breeding program during the period 2000–2018 and (ii) identify key agronomic traits contributing to the yield gains under various management conditions. Seventy-two early maturing hybrids developed by CIMMYT and three commercial checks were assessed under stress and non-stress conditions across 68 environments in seven eastern and southern African countries through the regional on-station trials. Genetic gain was estimated as the slope of the regression of grain yield and other traits against the year of first testing of the hybrid in the regional trial. The results showed highly significant (p< 0.01) annual grain yield gains of 118, 63, 46, and 61 kg ha^−1^ year^−1^ under optimum, low N, managed drought, and random stress conditions, respectively. The gains in grain yield realized in this study under both stress and non-stress conditions were associated with improvements in certain agronomic traits and resistance to major maize diseases. The findings of this study clearly demonstrate the significant progress made in developing productive and multiple stress-tolerant maize hybrids together with other desirable agronomic attributes in CIMMYT’s hybrid breeding program.

## Introduction

Maize (*Zea mays* L.) is a widely grown staple crop in Africa covering nearly 42 million hectares (ha) that accounting for 21% of the total global maize area. Africa’s total share of maize production, however, is 97 million tons, accounting only for approximately 8% of the world’s production (FAOSTAT, 2023). The crop is an important source of calories and protein for the poor households of Sub-Saharan Africa (SSA) (Shiferaw et al., 2011). Average annual maize consumption in Africa is 44 kg per capita yr^-1^, and it supplies 391 Kcal per capita day^-1^ and 10 g protein per capita day^-1^ (FAOSTAT, 2023). Maize consumption in Southern Africa is higher than the other regions, with food supply of 87 kg per capita yr^-1^ that represents 757 Kcal per capita day^-1^ and 20 g protein per capita day^-1^ (Shiferaw et al., 2011; FAOSTAT, 2023). Worldwide maize yields must double by 2050 to meet future needs (Ray et al., 2011). This will require an increase in the rate of yield gain from 1.6% yr^-1^ to 2.4% yr^-1^. From 1961 to 2021, maize production in Africa increased from 16 million metric tons to 97 million metric tons (FAOSTAT, 2023). This is a 6-fold increase in maize production across the continent. However, the increased production was mainly attributed to expansion in the area under maize production, as opposed to other regions of the world where an increase in maize production is largely associated with increased yields (Onyutha, 2019). Several studies indicated the potential of increasing maize productivity in Africa beyond the current levels through adoption of improved maize varieties and sustainable and intensive farming options (Abate et al., 2017; Cairns et al., 2021; Epule et al., 2022; Prasanna et al., 2022).

Low maize yield in most parts of Africa could be attributed to various factors, including inadequate adoption of climate-resilient varieties, suboptimal crop management, and environmental and socioeconomic conditions (Shiferaw et al., 2011). Maize is mainly grown with limited inputs under rainfed conditions by resource-limited farmers, often under the threat of diseases and insect pests (Mebratu et al., 2019; Prasanna et al., 2021). Low soil nitrogen (N), drought, and heat stress have long been recognized as the most important maize production constraints in Africa (Bänziger and Diallo, 2004; Diallo et al., 2004; Bänziger et al., 2006; Weber et al., 2012; Cairns et al., 2013). Under smallholder farmer conditions, these abiotic stresses can occur simultaneously, and their combined effect can cause a significant yield reduction (Cairns et al., 2021; Badu-Apraku et al., 2022). In addition to the inherent low soil fertility, low N stress is also due to removal of crop residues for use as animal feed and source of fuel, soil erosion/leaching, and poor weed control by the farmers (Bänziger et al., 2006). This situation may be worsened due to unavailability, inaccessibility, and unaffordability of fertilizers (Bonilla Cedrez et al., 2020). In addition, rainfall uncertainties and rise in temperature associated with climate change will further aggravate the intensity and frequency of drought in SSA (Shiferaw et al., 2014), increasing the vulnerability of smallholder farmers (Cairns et al., 2021). While drought can affect maize at all stages of growth and development, flowering and early grain-filling stages are the most sensitive, as drought stress disrupts pollination and reduces grain filling and kernel development (Bänziger et al., 2006; Edmeades et al., 2017). To alleviate the negative impacts of these stress factors, the development and deployment of multiple stresstolerant maize varieties are an important component of strategies to improve food security and income of smallholder farmers who mainly depend on maize for their livelihoods.

CIMMYT maize breeding programs in Eastern and Southern Africa (ESA), in close collaboration with various public and private sector institutions, have been engaged in the development and deployment of multiple stress-tolerant varieties (Cairns and Prasanna, 2018; Prasanna et al., 2021). Results of on-farm trials under low input and drought stress conditions showed that new stress-tolerant maize in ESA yields up to 25% more than the current commercial varieties (Setimela et al., 2017). Moreover, the yield potential of such varieties is not compromised under optimal growing conditions in climatically good years (Setimela et al., 2017; Cairns and Prasanna, 2018).

Estimating the rate of genetic gain within a breeding program provides an opportunity not only to monitor breeding efficiency and genetic progress (Eriksson et al., 2018) but also to identify gaps and steps toward improvement strategies for more effective breeding programs (Lee and Tollenaar, 2007). Genetic trends can be estimated using historical trial data or era studies, whereby varieties released in different years are evaluated in common trials. “Era studies” provide the most unbiased estimates of genetic gain because they avoid differences in agronomic management or climate variability that can confound the genetic trend. When genetic trends are estimated using historical data, nongenetic trends can only be analyzed for data that come from long-term trials conducted across years. In historical data, time trends are incorporated to show differences associated with historic variation in climate and crop management practices. All of the studies that dissected genetic gains into nongenetic and genetic components used historical trial data (e.g., Mackay et al., 2011; Laidig et al., 2014; Piepho et al., 2014; Laidig et al., 2017; Kumar et al., 2021; Laidig et al., 2021; Hartung et al., 2023; Rahman et al., 2023; Raymond et al., 2023). Using historical data collected over at least 10 years, these studies analyzed the contribution of genotypes (genetic trend) and of the environments (nongenetic trends) to quantify the impact of plant breeding and environmental factors to grain yield (GY) improvement over time. In the era studies, varieties from different years are tested in the same environment (e.g., optimal, managed drought, managed low N, and random stress) and year, management practices and time. This approach avoids differences in agronomic management or climate change confounding the genetic trend (Duvick, 2005; Badu-Apraku et al., 2017; Rutkoski, 2019, Badu-Apraku et al., 2021; Badu-Apraku et al., 2022; Masuka et al., 2017a, Masuka et al., 2017b; Liu et al., 2021; Asea et al., 2023). An era study was used for the first quantification of genetic gain within the maize breeding program in ESA at CIMMYT over the period 2000–2010 (Masuka et al., 2017a). Genetic gains for GY in the hybrid breeding program under optimal conditions, managed drought, random drought, low N, and maize streak virus (MSV) were estimated to have increased by 109.4 kg ha^−1^ yr^−1^, 32.5 kg ha^−1^ yr^−1^, 22.7 kg ha^−1^ yr^−1^, 20.9 kg ha^−1^ yr^−1^, and 141.3 kg ha^−1^ yr^−1^, respectively. In the open pollinated variety (OPV) maize breeding program, genetic gains for GY under optimal conditions, random drought, low N, and MSV were estimated to have increased by 109.9 kg ha^−1^ yr^−1^, 29.2 kg ha^−1^ yr^−1^, 84.8 kg ha^−1^ yr^−1^, and 192.9 kg ha^−1^ yr^−1^ in the early-maturity group and 79.1 kg ha^−1^ yr^−1^, 42.3 kg ha^−1^ yr^−1^, 53.0 kg ha^−1^ yr^−1^, and 108.7 kg ha^−1^ yr^−1^ in the intermediate–late maturity group (Masuka et al., 2017b). Using historical data from 2013 to 2021, Prasanna et al. (2022) reported genetic trends across CIMMYT’s tropical maize breeding pipelines globally. In the early-maturity breeding pipeline for Southern Africa, genetic trends were 138 kg ha^-1^ yr^-1^ (1.99% yr^-1^) under optimal conditions, 45 kg ha^-1^ yr^-1^ (2.13% yr^-1^) under managed drought, and 108 kg ha^-1^ yr^-1^ (2.87% yr^-1^) under random stress. There was no significant trend in GY under low N stress. Badu-Apraku et al. (2022) reported annual genetic gains in GY of 75 kg ha^−1^ yr^−1^ (2.91%) and 55 kg ha^−1^ yr^−1^ (1.33%) under low- and high-N environments, respectively. Era study conducted in Ethiopia showed genetic gain in GY of 62.26 kg ha^−1^ yr^−1^ (1.24% yr^−1^) in varieties released between 1973 and 2015; however, this study combined varieties ranging from old OPVs to new hybrids (Kebede et al., 2020). Asea et al. (2023) compared genetic trends of National Agricultural Research Organization (NARO)-Uganda, CIMMYT, and private seed companies’ hybrids released between 1999 and 2020 in Uganda and reported genetic gains of 1.30% yr^−1^ (59 kg ha^−1^ yr^−1^), 1.98% yr^−1^ (106 kg ha^−1^ yr^−1^, and 1.71% yr^−1^ (79 kg ha^−1^ yr^−1^), respectively.

**Figure 1:**
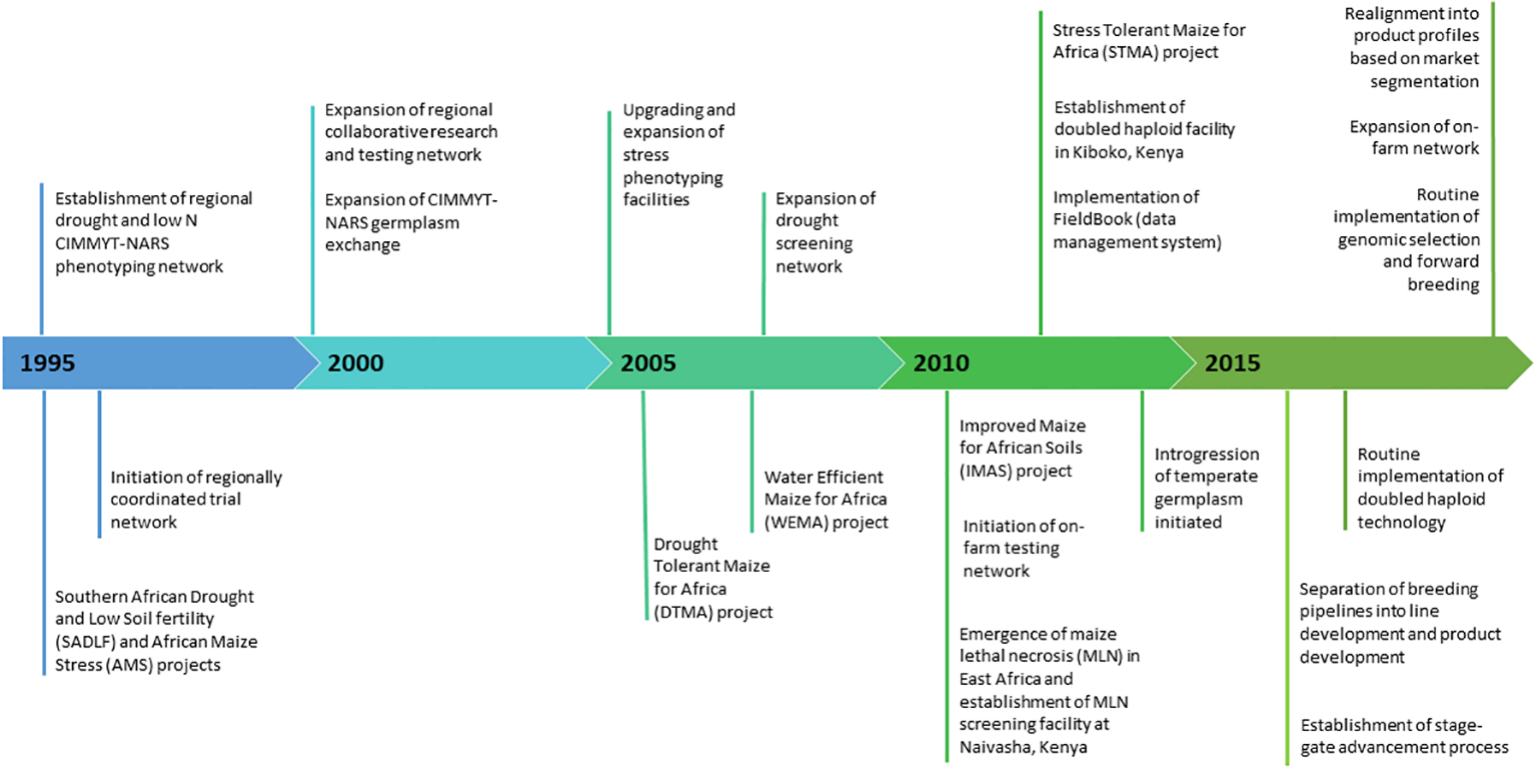
Key milestones in CIMMYT’s Southern African early-maturity maize breeding pipeline over the past two decades.

The early-maturity white maize market segment accounts for approximately 3.7 million hectares (M ha) in Southern Africa (including Malawi, Mozambique, South Africa, Zambia, and Zimbabwe), with an annual production of 5.0 million metric tons (MMT) and an estimated value of US$ 1.12 billion (https://ebs.excellenceinbreeding.org/wp-content/uploads/2023/03/MS-public.html). The market segment covers almost 2 million people and is largely comprising female-headed households (Cairns et al., 2022). The pipeline has undergone significant changes over the past 25 years (**Figure 1**). In 2006, increased donor investment allowed the expansion of the abiotic phenotyping network and regional trial network. In 2016, breeding pipelines moved from trait-based (project-based) to genetic gain breeding and transitioned into product profile by incorporating market intelligence. Over the past decade, the early-maturity breeding pipeline was modernized by wide adoption of electronic data capture (FieldBook), doubled haploid (DH) technology, forward breeding for MSV and genomic selection for GY under drought tolerance (Prasanna et al., 2021; Prasanna et al., 2022).

Masuka et al. (2017a) previously estimated genetic trends in southern Africa up to 2010; however, the newest hybrids in this study were developed in 2006. Given the extensive changes made in the CIMMYT Southern African early maize breeding pipeline over the past decade, it is important to reevaluate genetic progress. Thus, this study was conducted to (i) assess the genetic gains in GY of early maturing maize hybrids developed during 2000–2018 by the CIMMYT-Southern African early maturing maize breeding program and (ii) identify key agronomic traits that have contributed to the genetic progress of GY under various stress and non-stress conditions.

## Materials and methods

### Germplasm

An era panel was assembled from CIMMYT Southern African early maturing maize breeding program (<70 days to anthesis under normal growing conditions at CIMMYT-Harare Breeding Station) between 2000 and 2018. The hybrids were selected based on superior performance in the regional trials conducted across ESA and organized by the year they were first tested in the regional trials. A total of 75 hybrids, including 72 test hybrids and three benchmark commercial check hybrids, were used in the study. The number of hybrids tested per year ranged from 2 to 4, except for 2014, 2016, 2017, and 2018, where 5, 6, 6, and 7 hybrids, respectively, were included. Almost all of the hybrids tested were three-way crosses developed from fixed inbred lines that were generated through pedigree breeding or DH technology. The inbred line parents were selected through a series of testcross performance across stress and non-stress conditions from Stage 1 to Stage 3 trials. In addition, *per se* performances of the lines were assessed for yield performance, stress tolerance, and combining other desirable agronomic traits during the process of line development as well as in organized inbred line performance field trials. The breeding schemes adopted in generating the inbred lines and hybrids used in this study were described in detail by Prasanna et al. (2022). The three benchmark commercial check hybrids used were SC403 (released in 1998), SC513 (released in 1999), and Pan413 (released in 1998) that represented early maturing hybrids widely grown in ESA.

### Trial management

**Figure 2:**
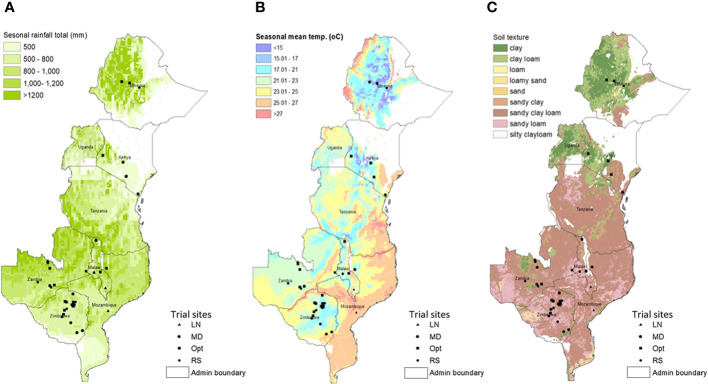
Spatial variability of **(A)** seasonal rainfall total (mm), **(B)** seasonal mean temperature (°C), and **(C)** soil texture in Eastern and Southern Africa regions with overlay of testing sites and management conditions used for the study.

A total of 68 trials, each consisting of a complete set of 75 hybrids, were planted during 2018–2019 across seven ESA countries, namely, Ethiopia, Kenya, Malawi, Mozambique, South Africa, Zambia, and Zimbabwe (**Table 1**). The trials were conducted at the experimental sites under management conditions that were representative of the maize-growing ecologies of the SSA (Hartkamp et al., 2000). These included 32 optimal (well-watered and well-fertilized), 14 low N stress, nine managed drought stress, and 13 environments that have undergone random stresses, including abiotic and biotic stresses (**Table 1**). Random stress is a mixture of several uncontrolled stress conditions that have happened intermittently at any stage during crop growing season affecting the yielding potential of the crop (Setimela et al., 2017; Prasanna et al., 2022). Geographical locations, weather, and soil parameters of the test locations are described in **Figure 2** and **Supplementary Table S1**. All of the optimally managed and low soil N and random stress trials were implemented during the respective main growing seasons of each country, whereas the managed drought stress trials were conducted on-station during the dry or winter seasons entirely under irrigation. Fertilizer rates were applied based on site-specific recommendations. The optimal trial sites were managed through crop rotation and incorporation of residues to maintain good soil health. All low N trials were planted on N-depleted plots and received no N fertilizer. Nitrogen depletion was achieved by continuously planting maize on the same plot for at least 5 years without N fertilizer application and by removing residues at harvest. Drought stress was induced by withholding irrigation 2 weeks ahead of anticipated flowering date through grain maturity. The targets of managed low N and drought stress experiments were to achieve 30%–40% of the average GY under optimum management condition at the same location (Bänziger et al., 2000; Menkir et al., 2022). Random stress trials were planted under rainfed conditions during the main growing seasons with suboptimal management at locations often experiencing simultaneous occurrence of several biotic and abiotic stresses including drought, pests, and diseases (Setimela et al., 2017; Prasanna et al., 2022). The hybrids were hand-planted in two-row plots of 4.0 m long with spacing of 0.75 m between rows and 0.25 m between plants and a final plant density of 53,333 plants ha^-1^. Initially, two seeds per hill were sown and then thinned to one seedling per hill 3 weeks after emergence. An alpha-lattice experimental design was used with three replications per entry.

**Table 1:** Summary of test locations by country for different trial management conditions in the present study.

Management	Ethiopia	Kenya	Malawi	Mozambique	South Africa	Zambia	Zimbabwe	Total
Optimum	2	4	2	1	2	4	17	32
Low nitrogen stress	2	2	2	1	2	1	4	14
Managed drought	–	2	–	–	–	–	7	9
Random stress	1	–	–	–	–	6	6	13
Total	5	8	4	2	4	11	34	68

### Measurements

Grain weight was measured from all of the shelled ears of each plot, and the percentage grain moisture content was determined. GY was calculated from the grain weight, adjusted to moisture content of 12.5%, and expressed in tons ha^-1^. In the low N and drought stress trials, data were collected from well-bordered plants by eliminating the plant nearest to the alley of each row to avoid border effects. Days to anthesis (AD) and silking (SD) were captured as the number of days from planting to when 50% of the plants had shed pollen and silk emergence, respectively. Anthesis–silking interval (ASI) was recorded as the difference between SD and AD. Nearly 2 weeks after silking, the averages of five representative plants were used to measure plant (PH) and ear (EH) heights as the distance from the soil level to the first tassel branch and the uppermost ear node, respectively. Root lodging (RL) and stem lodging (SL) were recorded as percentages of plants per plot that leaned more than 30° from vertical and had stems broken below the node bearing the upper ear, respectively. The number of ears per plant (EPP) was obtained by dividing the total number of ears harvested by the corresponding number of plants harvested. An ear was counted when it had at least one fully developed grain. Kernel texture (TEX) was rated using 1–5 scale, where 1 = flint and 5 = very dent. Bad husk cover (BHC) was recorded as a percentage of ears with exposed tips in a plot. Ear rot (ER) was expressed as a percentage of rotten ears to the total number of ears harvested per plot. Plant aspect (PA) was rated using a 1–5 scale as an overall phenotypic appearance of the plants per plot using visual assessment, where 1 = plots with uniform plants, good ear placement, big cob size, and less disease incident, and 5 = very poor overall appearance of the plants. Ear aspect (EA) was rated on a 1–5 scale, where 1 indicates large, well-filled, clean, and uniform ears, and 5 represents ears with undesirable characteristics using visual assessment. Leaf senescence (SEN) was scored for the low N and drought stress experiments on a scale of 1–10, where 1 indicates less than 10% the leaf area dead, and 10 indicates 100% dead leaf area (Bänziger et al., 2000). Harvest index (HI) was estimated at harvest as the ratio of GY to total aboveground biomass expressed in percentages. Diseases such as gray leaf spot (GLS), Turcicum leaf blight (TLB), and common rust (CR) were recorded on a scale of 1–9, where 1 = slight leaf infection and 9 = very severe leaf infection.

### Statistical analyses

Analysis of variance (ANOVA) for individual environment was carried out for GY and other traits using restricted maximum likelihood (REML) approach (Alvarado et al., 2020). Environments with heritability <0.2 were excluded from combined analyses. The analyses were conducted across all trials and separately for each management condition (optimal, low N, managed drought, and random stress). In each of these management conditions, site by year combinations were considered as environments. Variance components and best linear unbiased estimates (BLUEs) were determined for GY, other agronomic traits, and disease scores using a linear mixed model, treating test environment, replications, and incomplete blocks within replications as random and hybrids as fixed effects.


$${\text{Y}}_{\text{ijkl}}{\text{=µ+G}}_{\text{i}}{\text{+E}}_{\text{j}}{\text{+R}}_{\text{k}}\left({\text{E}}_{\text{j}}\right){\text{+B}}_{\text{l}}\left({\text{R}}_{\text{k}}{\text{E}}_{\text{j}}\right){\text{+GE}}_{\text{ij}}{\text{+ Ɛ}}_{\text{ijkl}}$$

where Y_ijkl_ is the performance of the i^th^ hybrid at j^th^ environment in the k^th^ replication of the l^th^ incomplete block; μ is the overall mean; G_i_ is the effect of the i^th^ hybrid; E_j_ is the effect of the j^th^ environment; R_k_(E_j_) is the effect of the k^th^ replication in the j^th^ environment; B_l_(R_k_E_j_) is the effect of l^th^ incomplete block nested into the k^th^ replication in the j^th^ environment; GE_ij_ is the ij^th^ hybrid × environment interaction effect; and Ɛ_ijkl_ is the residual effect. Broad sense heritability (*H*) for individual experiment with r replications was calculated as follows:


$$H=\frac{\delta_{g}^{2}}{\delta_{g}^{2}+\frac{\delta_{e}^{2}}{r}}$$

Heritability for measured traits was estimated across each management condition as follows:


$$H=\frac{\delta_{g}^{2}}{\delta_{g}^{2}+\delta_{\frac{g\times e}{e}}^{2}+\delta_{\frac{e}{re}}^{2}}$$

where *δ^2^_g_* represents hybrid variance, *δ^2^_g×e_* is the variance due to hybrid by environment interactions, *δ^2^* e is the error variance, e is the number of test environments, and r is the number of replications within a test environment.

A multi-environment trial analysis (META-R) package in the R software (Alvarado et al., 2020) was used to analyze phenotypic and genetic correlation coefficients among pairs of stress and non-stress environments. Adjusted mean GY and other measured traits of the hybrids developed over the 19-year period were used to estimate the genetic gain. As described by Khanna et al. (2022), the genetic gain was estimated separately for the four management conditions, *viz.*, (a) optimum, (b) low N stress, (c) managed drought stress, and (d) random stress by regressing trait mean against the year of first testing of the hybrid in the regional/advanced trial. Data from the commercial checks were excluded from the regression analysis. The genetic gain was represented by the regression coefficient (*b* value); mean GY and other traits of the hybrid were dependent variables (y); and the year of first testing of the hybrid was an independent variable (x). A genetic gain was declared significant when the probability of the regression coefficient was less than 0.05. The regression model used for the estimation of genetic gain was as follows:


$${\text{y}}_{ip}=\text{a}+{\text{bx}}_{i}{\text{ + ε}}_{\text{ip}}$$

where y_ip_ represents the adjusted mean of the i^th^ genotype first tested in p^th^ year, a is intercept, *b* is linear regression coefficient (genetic gain expressed in t ha^−1^y^−1^), x_i_ is year of first testing of the hybrid in the regional/advanced trial, and Ɛip is experimental error plus deviation from the regression model.

The genetic gain per annum was estimated by dividing the b value as the numerator by the intercept as the denominator and multiplied by hundred (Badu-Apraku et al., 2021) as follows:


$${\text{gg yr}}^{-1}=\frac{b}{a}\times 100$$

where gg yr^-1^ is genetic gain per year, b is linear regression coefficient, and a is intercept.

The statistical method used for genetic gain analysis in this era trial was selected based the genotypes studied and structure of the data. In this trial, breeding materials from different years were evaluated in a common set of environments that would overcome the confounding effects of changes in agronomic practices or climate change that cause nongenetic trends (Piepho et al., 2014; Rutkoski, 2019; Kumar et al., 2021; Rahman et al., 2023). Thus, the genetic trends in this dataset are due to breeding efforts, which can be assessed based on the year a genotype first entered the regional trial.

## Results

### Broad-sense heritability and variance components

Across optimum management conditions, heritability among all of the measured traits ranged from 0.13 for CR to 0.99 for AD, and most traits had high heritability of >0.60 except CR (**Table 2**). Under low N stress, heritability among measured traits ranged from 0.25 to 0.96 (**Table 3**). Traits such as GY, AD, SD, ASI, PH, EH, and TEX had high heritability ranging between 0.69 and 0.96, whereas the remaining traits showed low to moderate heritability of 0.25 to 0.56. Similarly, heritability under managed drought stress ranged from 0.22 for ASI to 0.96 for AD (**Table 4**). Several traits assessed under this stress had high heritability, except ASI (0.22), PA (0.49), and SL (0.40). Under random stress condition, heritability ranged from 0.32 (EPP) to AD (0.94). Most traits, including GY, AD, SD, PH, EH, TEX, and PA had high heritability of >0.85 (**Table 5**). Overall, heritability values for most traits were higher under optimum management conditions as compared to stress environments. For instance, GY had the highest heritability of 0.97 under optimum management, but heritability of 0.89, 0.88, and 0.84 under low N, managed drought, and random stress, respectively. In each environment, GY heritability ranged from 0.42 to 0.92 with a mean of 0.70 under optimum environments. Under each stress environment, heritability ranged from 0.20 to 0.73 (mean = 0.50), 0.56 to 0.79 (mean = 0.68), and 0.20 to 0.69 (mean = 0.46) under low N, managed drought, and random stress, respectively.

**Table 2:** Mean and genetic parameters for grain yield and other agronomic traits of early maturing maize hybrids evaluated under optimum conditions across 32 environments in Eastern and Southern Africa.

Mean/ variance	GY^+^	AD	SD	ASI	PH	EH	RL	SL	EPP	TEX	BHC	ER	HI	PA	EA	GLS	CR	TLB
Heritability	0.97	0.99	0.98	0.9	0.98	0.98	0.77	0.63	0.93	0.98	0.78	0.79	0.78	0.68	0.9	0.82	0.13	0.71
$\sigma_{ɡ}^{2}$	0.77**	4.21**	3.62**	0.33**	161.49**	97.03**	5.17**	2.26**	0.001**	0.25**	7.91**	2.88**	14.95**	0.05**	0.04**	0.51**	0.01	0.22**
$\sigma_{ɡxe}^{2}$	0.29**	0.63**	0.61**	0.20**	23.69**	19**	10.11**	6.77**	0.001**	0.03**	18.39**	8.43**	7.05**	0.08**	0.04**	0.66**	0.34**	0.35**
$\sigma_{e}^{2}$	1.86**	31.9**	38.26**	0.9**	764.2**	224.02**	8.49**	39.84**	0.01**	0.14**	16**	22.22**	73.32**	0.15**	0.12**	1.37**	2.01**	0.75**
$\sigma_{resid}^{2}$ .	24	2.13	2.21	1.7	177.97	145.1	38.4	36.73	0.02	0.16	47.41	21.09	40.44	0.23	0.14	1.11	0.6	0.76
Mean	7.77	65.1	66.39	1.22	233.35	117.45	3.79	4.06	1.09	2.77	6.1	5.98	41.82	2.66	2.82	3.51	2.37	3.55
Minimum	5.73	59.53	59.87	-0.16	184.8	90.48	0.96	1.12	1.00	1.62	1.16	2.37	29.03	1.91	2.36	2.03	1.71	2.31
Maximum	9.59	69.71	70.38	3.2	257.15	137.16	13.69	10.25	1.3	3.93	14.9	14.31	54.8	3.4	3.28	5.37	3.12	5.24
LSD	0.42	0.66	0.68	0.54	4.71	4.22	3.52	3.39	0.05	0.22	4.24	2.47	5.7	0.45	0.18	0.95	0.73	0.84
CV	14.3	2.2	2.2	107.3	5.7	10.3	163.6	149.3	12.1	14.4	112.8	76.7	15.2	18	13.4	30.1	32.7	24.6
#Locs	32	25	24	21	31	31	15	14	24	14	15	20	5	6	22	9	8	7

^+^GY, grain yield (t ha^-1^); AD, days to anthesis (d); SD, days to silking (d); ASI, anthesis–silking interval (d); PH, plant height (cm); EH, ear height (cm); RL, root lodging (%); SL, stalk lodging (%); EPP, number of ears per plant; TEX, kernel texture (1–5); BHC, bad husk cover (%); ER, ear rot (%); HI, harvest index (%); PA, plant aspect (1–5); EA, ear aspect (1–5); GLS, gray leaf spot (1–9); CR, common rust (1–9); TLB, Turcicum leaf blight (1–9). **Significant at P≤ 0.01.

**Table 3:** Means and genetic parameters for grain yield and other agronomic traits of early maturing maize hybrids evaluated under low nitrogen stress across 13 environments in Eastern and Southern Africa.

Mean/ variance	GY^+^	AD	SD	ASI	PH	EH	RL	SL	TEX	BHC	ER	EA	PA	SEN
Heritability	0.88	0.96	0.91	0.69	0.94	0.92	0.56	0.25	0.86	0.38	0.36	0.49	0.48	0.32
$\sigma_{ɡ}^{2}$	0.18**	5.00**	5.06**	0.24**	96.47**	41.12**	6.34**	2.66	0.15**	7.84*	1.87*	0.01**	0.01**	0.01*
$\sigma_{ɡxe}^{2}$	0.08**	1.46**	1.8**	0.35**	12.57**	12.28**	6.71**	9.51*	0.03*	18.91**	2.24*	0.03**	0.01*	0.04**
$\sigma_{e}^{2}$	0.52**	84.14**	36.16**	1.02**	814.67**	431.2**	0.001	26.01**	0.29**	235.32**	1.58**	0.15**	0.18**	0.24**
$\sigma_{resid}^{2}$ .	62	4.10	9.24	2.26	184.83	92.77	56.62	93.25	0.42	100.08	33.26	0.17	0.09	0.15
Mean	3.46	72.1	72.7	2.05	184.4	89.8	5.1	8.8	2.18	12.2	4.6	3.23	2.81	2.41
Minimum	2.36	67.1	66.5	0.75	145.9	73.3	0.5	3.0	1.39	3.5	0.2	2.91	2.45	1.96
Maximum	4.22	77.0	77.7	3.75	200.9	105.1	21.3	21.4	3.14	23.6	10.8	3.7	3.5	2.97
LSD	0.45	1.33	1.98	0.94	7.06	5.54	6.34	8.24	0.44	10.19	5.13	0.35	0.37	0.49
CV	22.7	2.8	4.2	73.2	7.4	10.7	146.7	110.1	29.6	81.7	125.6	12.9	11	16.2
#Locs	12	13	10	10	13	12	5	5	7	4	4	6	3	3

^+^GY, grain yield (t ha^-1^); AD, days to anthesis (d); SD, days to silking (d); ASI, anthesis–silking interval (d); PH, plant height (cm); EH, Ear height (cm); RL, root lodging (%); SL, stalk lodging (%); EPP, number of ears per plant; TEX, kernel texture (1–5); BHC, bad husk cover (%); ER, ear rot (%); HI, Harvest index (%); PA, plant aspect (1–5); EA, ear aspect (1–5); SEN, leaf senescence (1–10). *, ** Significant at *P* ≤ 0.05 and 0.01. respectively.

**Table 4:** Means and genetic parameters for grain yield and other agronomic traits of early maturing maize hybrids evaluated under managed drought across nine environments in Eastern and Southern Africa.

Mean/Variance	GY^+^	AD	SD	ASI	PH	EH	RL	SL	EPP	TEX	BHC	ER	EA	PA	SEN
Heritability	0.84	0.96	0.94	0.22	0.93	0.89	0.64	0.40	0.56	0.76	0.65	0.60	0.49	0.35	0.65
$\sigma_{ɡ}^{2}$	0.11**	4.07**	3.9**	0.05**	101.55**	66.32**	7.29**	1.36**	0.02**	0.08**	5.56**	16.91**	0.02**	0.03	0.08**
$\sigma_{ɡxe}^{2}$	0.11**	0.56**	0.78**	0.65**	21.7**	27.67**	15.64**	5.52**	0.03**	0.04**	4.41**	14.93**	0.03**	0.06**	0.04*
$\sigma_{e}^{2}$	0.53**	89.81**	94.29**	3.52**	613.96**	324.4**	62.98**	4.45**	0.09**	0.42**	1.6**	47.25**	0.78**	0.00	3.84**
$\sigma_{resid}^{2}$ .	27	3.07	4.38	1.84	139.81	132.35	40.13	32.97	0.01	0.17	23.38	55.00	0.17	0.17	0.51
Mean	2.62	68.8	70.0	1.17	200.7	104.5	8.0	4.5	0.78	3.18	3.6	13.7	3.69	3.2	5.93
Minimum	1.90	62.0	62.4	0.97	160.9	83.9	4.6	3.3	0.70	2.55	1.2	8.5	3.50	2.95	5.26
Maximum	3.27	73.0	74.1	1.42	221.5	119.4	17.5	6.6	0.83	3.66	10.3	32.5	3.90	3.54	6.58
LSD	0.4	1.35	1.53	0.56	8.34	8.00	4.67	2.55	0.08	0.39	3.96	7.45	0.26	0.4	0.48
CV	20	2.5	3	116.2	5.9	11	79.7	128.3	14.2	12.7	134	54.1	11.1	12.9	12
#Locs	9	9	9	7	9	9	7	8	5	4	4	3	5	2	5

^+^GY, grain yield (t ha^-1^); AD, days to anthesis (d); SD, days to silking (d); ASI, anthesis–silking interval (d); PH, plant height (cm); EH, ear height (cm); RL, root lodging (%); SL, stalk lodging (%); EPP, number of ears per plant; TEX, kernel texture (1–5); BHC, bad husk cover (%); ER, ear rot (%); HI, harvest index (%); PA, plant aspect (1–5); EA, ear aspect (1–5); GLS, gray leaf spot (1–9); CR, common rust (1–9); TLB, Turcicum leaf blight (1–9). **Significant at P≤ 0.01.

**Table 5:** Means and genetic parameters for grain yield and other agronomic traits of early maturing maize hybrids evaluated under random stress across nine environments in Eastern and Southern Africa.

Mean/variance	GY^+^	AD	SD	ASI	PH	EH	RL	SL	EPP	TEX	BHC	ER	EA
Heritability	0.89	0.94	0.93	0.71	0.91	0.91	0.55	0.51	0.32	0.92	0.51	0.45	0.65
$\sigma_{ɡ}^{2}$	0.24**	2.49**	2.45**	0.17**	86.84**	51.07**	7.95**	3.36**	0.001	0.12**	5.93**	6.26*	0.02**
$\sigma_{ɡxe}^{2}$	0.06**	0.96**	0.87**	0.19**	21.53**	9.0*	20.28**	4.12**	0.000	0.03**	11.73**	2.80	0.04**
$\sigma_{e}^{2}$	0.57**	35.96**	40.77**	0.34**	764.18**	427.83**	66.94**	3.33**	0.000	0.19**	16.9**	2.96	0.18**
$\sigma_{resid}^{2}$ .	63	1.77	2.10	1.12	280.78	161.55	96.10	35.43	0.01	0.10	49.35	37.68	0.21
Mean	4.30	65.7	66.6	1.18	190.5	88.76	9.7	4.7	1.02	2.45	6.0	8.6	2.93
Minimum	2.95	61.3	61.9	0.36	153.3	69. 9	1.4	0.8	0.86	1.72	1.0	1.6	2.60
Maximum	5.19	69.6	70.0	2.57	207.4	101.8	23.8	13.2	1.20	3.31	14.5	22.0	3.46
LSD	0.51	1.19	1.20	0.76	8.74	6.67	7.45	5.14	0.15	0.29	6.75	7.89	0.32
CV	18.5	2	2.2	89.2	8.8	14.3	101.2	127.6	11.7	12.8	118	71.6	15.6
#Locs	9	9	9	8	13	12	8	5	2	6	5	2	9

^+^GY, grain yield (t ha^-1^); AD, days to anthesis (d); SD, days to silking (d); ASI, anthesis–silking interval (d); PH, plant height (cm); EH, ear height (cm); RL, root lodging (%); SL, stalk lodging (%); EPP, number of ears per plant; TEX, kernel texture (1–5); BHC, bad husk cover (%); ER, ear rot (%); HI, harvest index (%); PA, plant aspect (1–5); EA, ear aspect (1–5); GLS, gray leaf spot (1–9); CR, common rust (1–9); TLB, Turcicum leaf blight (1–9). *, ** Significant at *P* ≤ 0.05 and 0.01. respectively.

The analyses of variance showed highly significant genotypic (G), environment (E), and G × E interaction effects for most studied traits under all management conditions, except for CR under optimum, SL under low N, and EPP under random stress environments (**Tables 2** **–** **5**). Variances due to environments were higher under all management conditions followed by genotypic variances, whereas G × E variances were very low. Across all trials, variances due to E and G × E were significantly higher than G variances, since the management conditions of the test environments were distinctly different from each another (**Supplementary Table S2**).

### Performances of era hybrids

Mean, minimum, and maximum performances of all traits measured under various management conditions were presented in **Tables 2** **–** **5** and **Supplementary Table S3**. Under optimum management (**Table 2**), mean GY was 7.77 t ha^-1^, with a range between 5.73 and 9.59 t ha^−1^. Under low N stress conditions, GY varied from 2.36 to 4.22 t ha^-1^, with a mean of 3.46 t ha^-1^ (**Table 3**). Mean GY was 2.62 t ha^-1^, with a range of 1.90–3.27 t ha^-1^ under managed drought environments (**Table 4**). Across random stress environments, GY ranged from 2.95 to 5.19 t ha^-1^, with a mean of 4.30 t ha^-1^ (**Table 5**). Among agronomic traits, AD, SD, ASI, ER, and percent of lodged plants increased under stress conditions as compared to optimum management, while significant reduction was observed in EPP under stress environments (**Tables 2**-**5**).

### Effects of stress environments on grain yield

**Figure 3:**
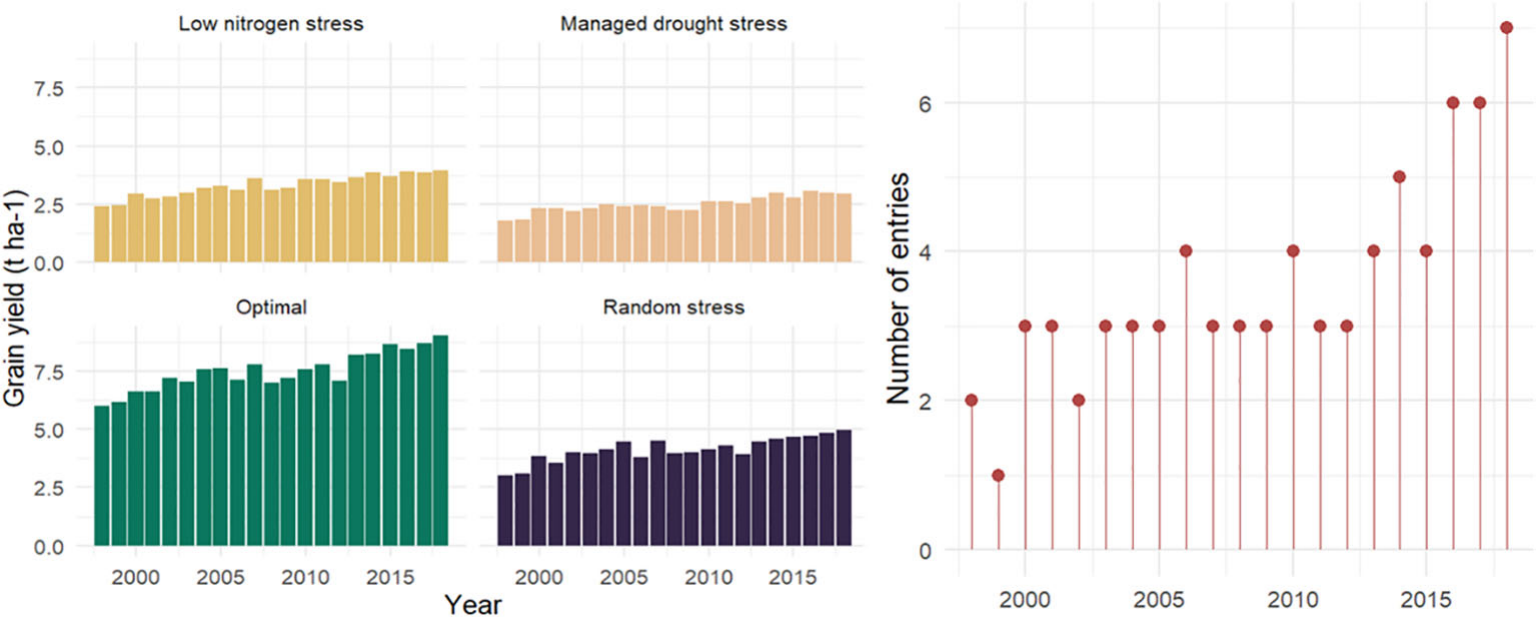
Mean grain yield and number of hybrids evaluated across contrasting environments in Eastern and Southern Africa during 2018–2019. Hybrids from the years 1998 and 1999 are commercial checks.

**Figure 4:**
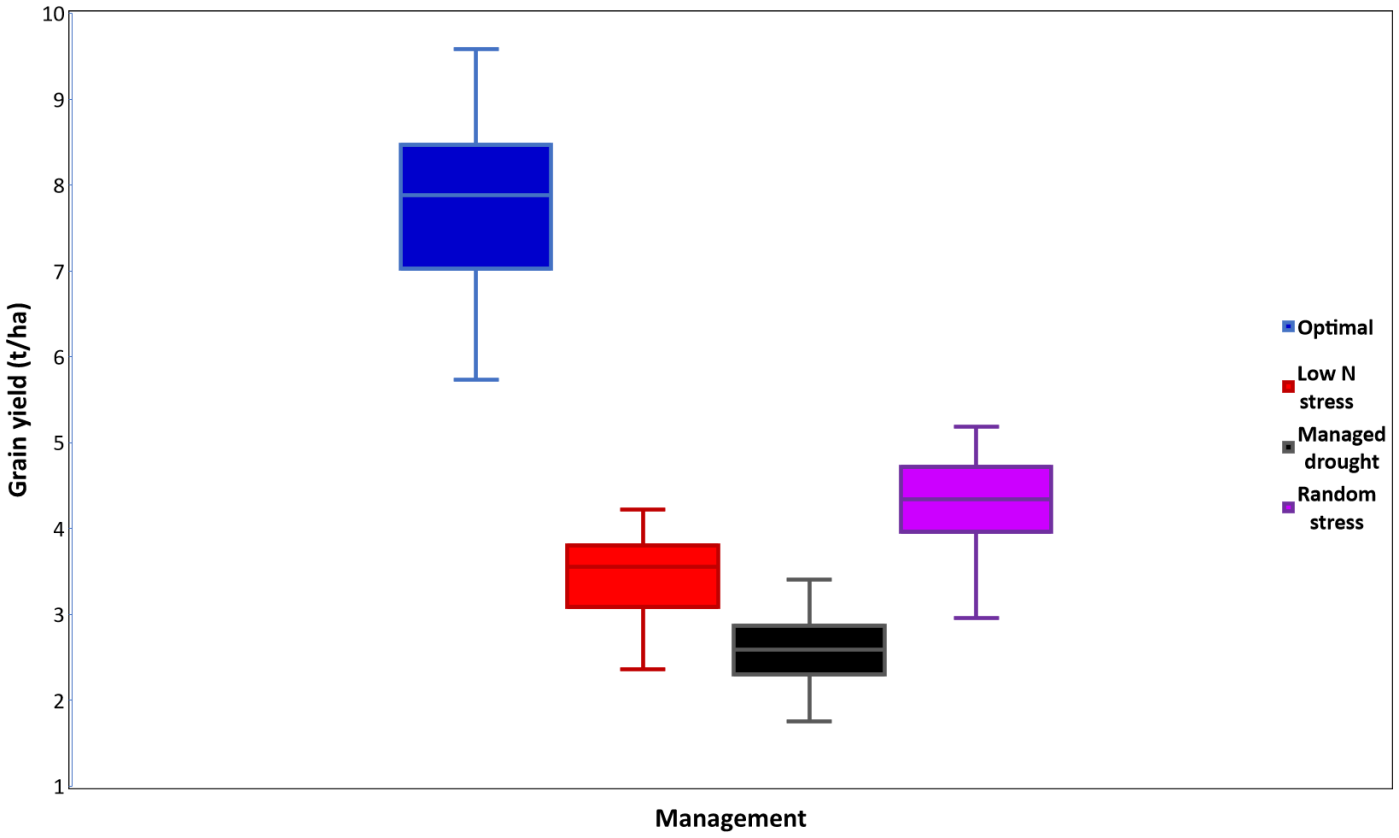
Mean grain yield (t ha^-1^) of era hybrids evaluated under optimal, low N, managed drought, and random stress management conditions in trials conducted in 2018 and 2019.

The era hybrids had higher GY than the commercial checks under all management conditions (**Supplementary Figure S1**). On average, the hybrids showed 26%, 40%, 41%, and 40% yield advantages over the mean of commercial checks under optimum, low N, managed drought, and random stress, respectively. The yield increases over the mean of benchmarked check hybrids ranged from 9% to 48% under optimal, 13% to 62% under low N, 21% to 70% under managed drought, and 17% to 62% under random stress conditions. Proportionally, the era hybrids had greater yield advantage over the checks under stress conditions than non-stress environments (**Supplementary Figure S1**). Hybrids selected in the later years, especially from 2013 to 2018, had higher GY advantage over the benchmarked commercial check hybrids (released in 1998 and 1999) than the hybrids selected during 2000 to 2012 (**Figure 3**). As compared to optimum management, low N stress environments used for this study reduced hybrid yields by an average of 56%, with a range of 51%–61%. Similarly, the mean GY reductions were 67% and 45% under managed drought and random stress, with ranges of 64%–70% and 42%–60%, respectively (**Figure 4**; **Supplementary Figure S2**). For GY, positive and significant correlation coefficients that ranged from 0.78** to 0.921** were observed among pairs of data combined within each management condition. Managed drought had positive and significant (*r* = 0.78**) correlation with optimum management and random stress. Low N stress environment had strong positive correlations (*r* > 0.80**) with the other management conditions. A strong and positive association (*r* = 0.91**) was observed between optimum and random stress environments (data not presented). However, analysis of correlation coefficients for GY among selected individual sites under stress and non-stress environments showed a strong association among optimum and random stress environments (**Supplementary Table S4**). On the other hand, low N and managed drought stress environments had a low correlation coefficient with most stress and non-stress environments.

### Genetic gains in grain yield and other measured traits under optimal and stress environments

**Figure 5:**
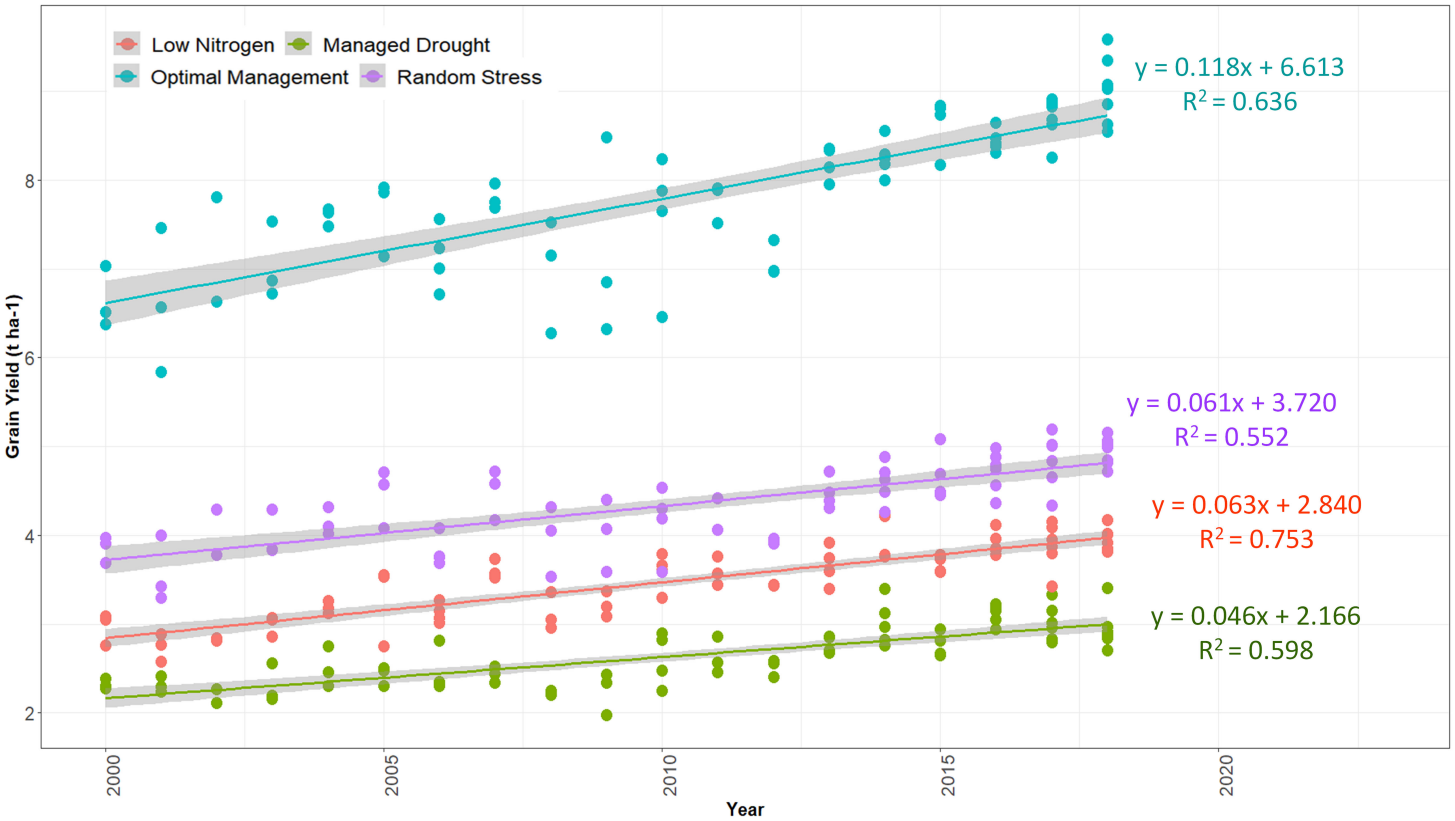
Genetic trends for grain yield in early maturing maize hybrids selected from 2000 to 2018. The hybrids were evaluated in Eastern and Southern Africa regions during 2018 and 2019 under optimum, low nitrogen stress, managed drought stress, and random stress conditions.

Significant improvements in GY and other traits were observed in the hybrids identified over the 19-year study period (2000–2018). The results of regression analyses showed annual GY increases of 118 kg ha^−1^ yr^−1^, 63 kg ha^−1^ yr^−1^, 46 kg ha^−1^ yr^−1^, and 61 kg ha^−1^ yr^−1^, indicating annual yield gains of 1.78%, 2.21%, 2.13%, and 1.64% under optimum, low N, managed drought, and random stress conditions, respectively (**Figure 5**; **Tables 6**–**9**). The coefficient of determination (R^2^) for hybrid GY regressed against the year of first testing in regional trials ranged from 0.55 to 0.75. Among the other measured traits, RL, SL, TEX, PA, GLS, and TLB had significant (p< 0.01) regression coefficients under optimal environment. RL and SL decreased by 3.0% and 2.5%, respectively. Kernel TEX score increased by 0.04 (1.71%) per year. Among the diseases, GLS and TLB scores decreased by 0.05 and 0.04, representing annual decreases of 1.27% and 1.02% in disease scores, respectively. There was no significant change in AD, SD, ASI, PH, EH, EPP, BHC, ER, HI, EA, and CR under optimal management (**Table 6**).

**Table 6:** Intercept, regression coefficients (b), relative genetic gain and coefficient of determination (R^2^) of grain yield, and other agronomic traits of early maturing maize hybrids evaluated under optimum environments in Eastern and Southern Africa.

Trait	Trait abbreviation	Intercept	*b*	Relative gain (% yr^-1^)	R^2^	p-value	SE+*b*
Grain yield (t ha^-1^)	GY	6.61	0.1176	1.78	0.6359	0.0000	0.0106
Days to anthesis (d)	AD	65.14	-0.0006	0.00	0.0000	0.9893	0.0439
Days to silking (d)	SD	66.44	-0.0036	-0.01	0.0001	0.9288	0.0407
Anthesis–silking interval (d)	ASI	1.19	0.0002	0.02	0.0000	0.9877	0.0127
Plant height (cm)	PH	229.2	0.4177	0.18	0.0346	0.1176	0.2636
Ear height (cm)	EH	116.4	0.1226	0.11	0.0048	0.5625	0.2107
Root lodging (%)	RL	5.18	-0.1546	-2.98	0.1449	0.0008	0.0442
Stalk lodging (%)	SL	5.41	-0.1353	-2.50	0.1607	0.0005	0.0370
Ears per plant (#)	EPP	1.06	0.0024	0.23	0.0378	0.1019	0.0015
Kernel texture (1–5)	TEX	2.33	0.0399	1.71	0.2097	0.0093	0.0094
Bad husk cover (%)	BHC	5.80	0.0179	0.31	0.0010	0.7886	0.0665
Ear rot (%)	ER	5.94	-0.0209	-0.35	0.0069	0.4880	0.0300
Harvest index (%)	HI	42.23	-0.0687	-0.16	0.0091	0.4258	0.0858
Plant aspect (1–5)	PA	2.90	-0.0240	-0.83	0.2306	0.0000	0.0052
Ear aspect (1–5)	EA	2.89	-0.0079	-0.27	0.0504	0.0580	0.0041
Gray leaf spot (1–9)	GLS	4.04	-0.0514	-1.27	0.1363	0.0014	0.0155
Common rust (1–9)	CR	2.44	-0.0074	-0.30	0.0216	0.2178	0.0060
Turcicum leaf blight (1–9)	TLB	3.96	-0.0403	-1.02	0.1661	0.0004	0.0108

**Table 7:** Intercept, regression coefficients (b), relative genetic gain and coefficient of determination (R^2^) of grain yield, and other agronomic traits of early maturing maize hybrids evaluated under low nitrogen stress environments in Eastern and Southern Africa.

Trait	Trait abbreviation	Intercept	*b*	Relative gain (% yr^-1^)	R^2^	p-value	SE+*b*
Grain yield (t ha^-1^)	GY	2.84	0.0629	2.21	0.7529	0.0000	0.0043
Days to anthesis (d)	AD	72.14	0.0004	0.00	0.0000	0.9931	0.0484
Days to silking (d)	SD	72.56	0.0105	0.01	0.0006	0.8345	0.0501
Anthesis–silking interval (d)	ASI	1.88	0.0131	0.70	0.0162	0.2866	0.0122
Plant height (cm)	PH	181.0	0.3576	0.20	0.0411	0.0878	0.2066
Ear height (cm)	EH	89.22	0.0700	0.08	0.0035	0.6222	0.1414
Root lodging (%)	RL	7.27	-0.2097	-2.89	0.1217	0.0027	0.0673
Stalk lodging (%)	SL	10.72	-0.1758	-1.64	0.0867	0.0120	0.0682
Kernel texture (1–5)	TEX	1.87	0.0282	1.51	0.1509	0.0007	0.0080
Bad husk cover (%)	HC	12.75	0.0599	0.47	0.0057	0.5288	0.0947
Ear rot (%)	ER	3.61	0.0730	2.02	0.0393	0.0950	0.0432
Plant aspect (1–5)	PA	3.22	-0.0003	-0.01	0.0001	0.9345	0.0033
Ear aspect (1–5)	EA	2.92	-0.0116	-0.40	0.1461	0.0009	0.0033
Senescence (1–10)	SEN	2.41	0.0004	0.02	0.0001	0.9360	0.0044

**Table 8:** Intercept, regression coefficients (b), relative genetic gain and coefficient of determination (R^2^) of grain yield, and other agronomic traits of early maturing maize hybrids evaluated under managed drought stress environments in Eastern and Southern Africa.

Trait	Trait abbreviation	Intercept	*b*	Relative gain (% yr^-1^)	R^2^	p-value	SE+*b*
Grain yield (t ha^-1^)	GY	2.17	0.0462	2.133	0.5975	0.0000	0.0045
Days to anthesis (d)	AD	68.55	0.0185	0.027	0.0025	0.6749	0.0439
Days to silking (d)	SD	69.77	0.0208	0.030	0.0033	0.6309	0.0430
Anthesis–silking interval (d)	ASI	1.02	0.0138	1.359	0.0254	0.1815	0.0102
Plant height (cm)	PH	196.4	0.4124	0.210	0.0498	0.0596	0.2154
Ear height (cm)	EH	103.6	0.0933	0.090	0.0036	0.6142	0.1843
Root lodging (%)	RL	8.87	-0.0842	-0.949	0.0191	0.2469	0.0722
Stalk lodging (%)	SL	5.26	-0.0826	-1.569	0.0630	0.0335	0.0381
Ears per plant (#)	EPP	0.75	0.0030	0.400	0.1014	0.0064	0.0011
Kernel texture (1–5)	TEX	3.01	0.0159	0.529	0.0822	0.0146	0.0064
Bad husk cover (%)	HC	3.15	0.0300	0.952	0.0035	0.6198	0.0602
Ear rot (%)	ER	12.86	0.0191	0.148	0.0009	0.8057	0.0774
Plant aspect (1–5)	PA	3.67	0.0007	0.019	0.0005	0.8573	0.0037
Ear aspect (1–5)	EA	3.24	-0.0063	-0.194	0.0165	0.2816	0.0058
Senescence (1–10)	SEN	6.06	-0.0096	-0.159	0.0264	0.1727	0.0070

**Table 9:** Intercept, regression coefficients (b), relative genetic gain and coefficient of determination (R^2^) of grain yield, and other agronomic traits of early maturing maize hybrids evaluated under random stress environments in Eastern and Southern Africa.

Trait	Trait abbreviation	Intercept	*b*	Relative gain (% yr^-1^)	R^2^	p-value	SE+*b*
Grain yield (t ha^-1^)	GY	3.72	0.0608	1.635	0.5523	0.0000	0.0065
Days to anthesis (d)	AD	65.73	-0.0018	-0.003	0.0000	0.9578	0.0348
Days to silking (d)	SD	66.50	0.0111	0.017	0.0015	0.7479	0.0343
Anthesis–silking interval (d)	ASI	1.03	0.0126	1.227	0.0216	0.2181	0.0101
Plant height (cm)	PH	187.9	0.2694	0.143	0.0240	0.1938	0.2053
Ear height (cm)	EH	88.35	0.0557	0.063	0.0017	0.7282	0.1597
Root lodging (%)	RL	11.79	-0.2114	-1.793	0.0948	0.0085	0.0781
Stalk lodging (%)	SL	5.92	-0.1220	-2.059	0.0697	0.0251	0.0533
Ears per plant (#)	EPP	1.02	0.0006	0.058	0.0031	0.6403	0.0013
Kernel texture (1–5)	TEX	2.17	0.0260	1.198	0.1680	0.0003	0.0069
Bad husk cover (%)	HC	4.60	0.1033	2.243	0.0331	0.1261	0.0667
Ear rot (%)	ER	7.83	0.0235	0.300	0.0021	0.7015	0.0610
Ear aspect (1–5)	EA	2.90	0.0000	0.001	0.0000	0.9904	0.0035

Under low N stress, significant (p< 0.05) changes in the performance of hybrids were observed for RL, SL, TEX, and PA but nonsignificant for all other traits (**Table 7**). Percent change in RL and SL over the study period was -2.9% and -1.6%, respectively. Kernel TEX score showed an annual increase of 1.5%, whereas PA score decreased by 0.01 per year, denoting a yearly decrease of 0.40%. Under managed drought, regression analyses showed significant changes in SL, EPP, and TEX only but not for the other traits (**Table 8**). Annually, SL decreased by 1.6%, whereas EPP and TEX increased by 0.40% and 0.53%, respectively. Under random stress, only RL, SL, and TEX had significant regression coefficient of -0.21, -0.12, and 0.03, representing relative annual changes of -1.79%, -2.06%, and 1.20%, respectively.

## Discussion

The current study was conducted to assess the genetic gains of hybrids developed during a period of 19 years (2000–2018) by CIMMYT-Southern Africa early-maturity white maize breeding program. Most measured traits had high broad-sense heritability (>0.60) under all management conditions, indicating that these traits are highly heritable and selection for improvement of the traits would be effective. However, heritability values under stress environments were lower than that of optimal, which attributed to high residual variances in the stress trials (Weber et al., 2012; Masuka et al., 2017a; Masuka et al., 2017b; Das et al., 2019). As shown in **Tables 2** **–** **5**, residual variances for GY under low N and managed drought stress were 2.9 and 4.7 times higher than genetic variances under the same management. Under optimum management and random stress conditions, however, residual variances for the same trait were 2.4 times that of genetic variance. The higher heritability of GY and other measured traits under optimum conditions implied greater genetic variance under optimum conditions compared to stress environments, suggesting the efficiency of selection for these traits under optimum conditions. Similar to the current findings, Bänziger et al. (1997) and Ertiro et al. (2022) reported 29% and 30%, respectively, less heritability under low N as compared to optimum conditions.

Significant genotypic variances for GY and most measured traits in both the stress and non-stress environments indicate the presence of considerable genetic variability among the era hybrids studied. This suggests that further genetic gains from selection can be achieved for improvements in GY and other studied traits under the target environments. Significant environmental variances for almost all of the studied traits show that each test environment was unique in identifying superior hybrids. The significant G × E variances observed for GY and other traits under stress and optimal environments indicated inconsistent expression of the traits and change in the ranking of the era hybrids across the test environments. This result signifies the need for extensive testing of the hybrids under different management conditions to identify hybrids with consistent performance. Similar findings were previously reported in an era study for maize hybrids evaluated in West Africa under stress and non-stress environments (Badu-Apraku et al., 2017; Badu-Apraku et al., 2022). Badu-Apraku et al. (2017) indicated that the variable response of genotypes to varying environmental conditions constitutes a major challenge in the identification of superior maize hybrids for wide or narrow adaptation. Thus, breeders need to devise a suitable breeding strategy to identify elite multiple stress-tolerant hybrids with stable performance across a targeted population of environments.

The hybrids used in this era study showed variable performances as depicted by hybrid means and ranges of values for various traits under stress and non-stress environments. Almost all of the hybrids had higher GYs and other desirable traits than the benchmark commercial check hybrids, indicating the potential for identifying and commercializing new sets of hybrids with superior performance over the commercial hybrids that were under production in ESA when the era hybrids under study were identified. Advantages of newly developed stress-tolerant hybrids over the commercial check hybrids in GY and other desirable traits have been previously reported by various investigators (Badu-Apraku et al., 2017; Setimela et al., 2017; Worku et al., 2020; Menkir et al., 2022). The era hybrids showed higher GY advantage over the commercial checks under stress conditions (40%–41%) than under optimal environmental conditions (26%), indicating the substantial progress made by CIMMYT, in collaboration with public and private sector institutions, in improving tropical maize germplasm for stress tolerance. The improvement has been mainly attributed to increased stress tolerance, primary drought, and low soil fertility tolerance (Bänziger et al., 2000; Bänziger et al., 2006; Setimela et al., 2017; Worku et al., 2020; Prasanna et al., 2021; Prasanna et al., 2022).

The yield reductions observed under the stress conditions used for this study, as compared to the optimal environments, were comparable to the 40%–60% average yield reductions reported by Bänziger et al. (2000) to characterize maize genotypes for stress tolerance. Similar to the current findings, Setimela et al. (2017) reported average GY reductions of 77%, 68%, and 54% under low N, managed drought, and random stress as compared to optimum management in regional yield trials conducted in ESA. Regional collaborative yield trials conducted for 8 years in West Africa showed that drought stress reduced average hybrid maize yields by 54%–80% (Menkir et al., 2022). Variable levels of GY reductions would be expected under managed and random stress conditions depending on the intensity of stress under which the crop is grown (Mebratu et al., 2019).

Comparative performances of the hybrids used in this study relative to the commercial checks were assessed in the regional trials conducted during 2000–2018. Commercial checks included in the regional trials and in this era trial were the best available hybrids widely grown in the ESA region. This study consisted of the best hybrids selected from the regional trials, with GY advantages of at least 10% or significantly better performance for other key traits as compared to the mean of the commercial checks in the regional/advanced trials. Prerelease maize hybrids selected based on regional performance data are announced on the CIMMYT website (www.cimmyt.org) for licensing by NARS and seed company partners that will register and commercialize the selected hybrids. Several hybrids from this era panel were registered and commercialized in one or more countries. For example, hybrids CZH1258, CZH142055, and CZH15467 (**Supplementary Table S3**) were commercialized each in more than five countries with different names.

Poor correlation of low N and managed drought stress environments with other stress and non-stress environments indicated that selection under one management condition for high GY performance was less efficient than selection under the target environmental condition. Therefore, maize breeding programs targeting stress and non-stress production conditions in SSA should include the desired selection environments to improve the selection efficiency (Bänziger et al., 1997; Ertiro et al., 2020). As suggested by Bänziger et al. (2006) and Weber et al. (2012), combined evaluations across optimum management, low N, managed drought, and random stress conditions might be of advantage for indirect selection. A relatively strong association among optimum and random stress environments (**Supplementary Table S3**) showed that greater improvements under random stress were predicted for direct selection and indirect selection under optimum management conditions.

Significant genetic gains of 118 kg ha^−1^ yr^−1^, 63 kg ha^−1^ yr^−1^, 46 kg ha^−1^ yr^−1^, and 61 kg ha^−1^ yr^−1^ were observed for GY under optimal, low N stress, managed drought stress, and random stress conditions, respectively, in the CIMMYT Southern Africa early-maturity breeding program over the past two decades. The yield gains were higher than those of most studies previously reported (**Table 10**). Particularly, higher genetic gain was observed under optimal management and low N stress as compared to the reports of Masuka et al. (2017a); Masuka et al., (2017b) and Prasanna et al. (2022) on CIMMYT’s breeding pipelines in ESA. This is, in part, due to the longer period this study covered, the methodology used, and genotypes studied. In this study, era hybrids selected from the southern African early maturing maize breeding pipeline regional trials conducted from 2000 to 2018 were evaluated in common trials across various management conditions. Prasanna et al. (2022) used historical data from 2013 to 2021 regional hybrid trials to monitor real-time genetic trends and provide a baseline for future investments in tropical maize breeding. Masuka et al. (2017a) evaluated era panel of 67 best-performing hybrids selected from the regional trials of CIMMYT ESA maize breeding pipelines conducted from 2000 to 2010 in eight locations across six countries. These hybrids were drawn from various breeding pipelines and were not disaggregated by maturity groups and adaptation agroecology. Masuka et al. (2017b) documented genetic gain for maize GY within the CIMMYT ESA OPV breeding pipeline using varieties selected from regional trials over a 12-year period (1999–2011).

**Table 10:** Estimates of genetic gain across various breeding programs under different management conditions.

Germplasm	Location^*^	Genetic gain (kg ha^-1^ yr^-1^)^**^					Time period	Methodology	Reference
		Optimal	Managed drought	Random stress	Low nitrogen	Multiple environments			
Extra-early hybrids	WA	140.25 (4.15)	46.54 (4.14)				2008-2016	Era study	Badu-Apraku et al. (2021)
Early hybrids	WA	75. (1.33)			55.0 (2.91)		2017-2019	Ear study	Badu-Apraku et al. (2022)
Hybrids	ESA	109.4 (1.4)	32.5 (0.85)	22.7 (0.85)	20.9 (0.62)		2000-2010	Era study	Masuka et al. (2017a)
Open pollinated varieties (OPVs)	ESA	109.9 (1.76)	24 (0.97)	29.2 (1.21)	84.8 (3.11)		2000-2010	Era study	Masuka et al. (2017b)
Intermediate hybrids	WA	62.65 (1.47)	11.89 (0.74)			56.11 (1.32)	2012-2019	Historical data	Menkir et al. (2022)
Early hybrids	SA	138 (1.99)	45 (2.13)	108 (2.87)			2013-2021	Historical data	Prasanna et al. (2022)
OPVs/hybrids	Uganda					81.0 (2.25)	2008-2020	Historical data	Asea et al. (2023)
Hybrids	Uganda					55.0 (1.69)	1999-2020	Era study	Asea et al. (2023)

^*^WA, West Africa; ESA, Eastern and Southern Africa; SA, Southern Africa.

^**^Number in brackets is rate of genetic gain expressed as a percentage.

Genetic gains in GY within CIMMYT’s Southern Africa early-maturity maize breeding program are likely to be, in part, associated with the significant donor investment over the past two decades. The continued investment in the breeding program has allowed expansion of the phenotyping network and use of innovative breeding tools and technologies. Expansion of testing networks from very few CIMMYT and NARS research stations in early 2000s to diverse on-station testing environments across ESA has led to improved selection accuracy for important traits. Some of the lines and testers used to constitute the recent maize hybrids included in this study were developed using DH technology, besides introgression of drought-tolerant, disease-resistant, and expired Plant Variety Protection (ex-PVP) temperate maize germplasm.

Although considerable gains in GY were observed in this study under various management conditions, actual yield gain was higher under optimum management (118 kg ha^−1^ yr^−1^) as compared to those of stress trials (46–63 kg ha^−1^ yr^−1^). Percent annual yield gains, on the other hand, were higher under low N (2.21% yr^−1^) and managed drought (2.13% yr^−1^) stresses than those under optimum management (1.78% yr^−1^) and random stress (1.64% yr^−1^) environments, indicating that the hybrids selected for the study had favorable alleles or allele combinations responsible for increased GY under non-stress conditions. In this study, the random stress environments consisted of seasonal drought, late planting, low soil fertility, and weed infestation. These environments accurately simulate the stress conditions experienced by smallholder farmers in SSA (Masuka et al., 2017a).

The significant gains in GY realized in this study under both stress and non-stress conditions were associated with improvements in certain agronomic traits and resistance to major diseases (GLS and TLB). Moreover, there was a significant reduction in SL and RL across all environments (with the exception of RL under drought stress). Both RL and SL are key traits used in the stage-gate advancement of candidate hybrids within CIMMYT’s Southern Africa early-maturity maize breeding program. Badu-Apraku et al., 2017; Badu-Apraku et al., 2022) also reported that genetic gain in GY was strongly associated with improved lodging resistance.

While the early-maturity maize market segment in Southern Africa contains both flint and dent (except in Malawi), we found a significant shift toward dent kernels. In Malawi, the market demands flint maize varieties, and low market penetration of new improved maize varieties was linked to the release of dent varieties (Lunduka et al., 2012). In most ESA countries, however, farmers prefer maize varieties with semi-flint texture (Kassie et al., 2017; Ekpa et al., 2018). Flint maize varieties are resistant to storage pests and provide higher flour output per unit of grain, while dent textured grains are softer and can easily be pounded compared to flint maize (Kassie et al., 2017). The shift toward dent kernel texture was due to indirect selection for improved GY, since maize genotypes with dent kernel texture tend to show higher GY than flint genotypes (Tamagno et al., 2015). Tamagno et al. (2015) observed that kernel number and weight were significantly higher in dents when compared to those of flints. The trend toward selection for dent type was also reported in the NARO (Uganda) breeding program (Asea et al., 2023).

There was no change in EPP in this study except under drought stress where the number of ears increased by 0.4% between 2000 and 2018. No significant change was observed in ASI since the hybrids used in this study have already undergone at least three successive stages of performance evaluation and selection under stress and non-stress conditions. Badu-Apraku et al., 2021; Badu-Apraku et al., 2022) also reported no change in ASI in early and extra early maturing hybrids developed during 2008–2016. There were no significant changes in DA and DS across environments. These are to be expected since days to flowering as indicators of maturity are key traits used in variety advancement and germplasm that falls outside the range would not be selected. PH and EH were not changed during the study period, indicating that the hybrids were selected for stable plant stature that is tolerant to lodging. Several reports on tropical maize germplasm indicated the lack of strong association between gain in GY and change in other agronomic traits, including maturity and plant stature (Badu-Apraku et al., 2021, Badu-Apraku et al., 2022; Masuka et al., 2017a). Masuka et al. (2017a) argued that yield gain is not a function of an increase in the length of the photosynthetic period associated with increased maturity but rather a direct increase in tolerance to multiple stresses and/or an increase in overall yield potential. Duvick (2005) found no change in plant and ear heights in temperate germplasm over 70 years. The results of the present study also showed no significant changes in HI, which might be due to lack of direct selection for the trait in the breeding process. Despite the importance of HI for productivity, limited attention has been given to this trait in tropical maize breeding. Ruiz et al. (2023) provided the most comprehensive assessment of maize HI evolution over years of commercial maize breeding programs of temperate germplasm. The study indicated little or no increase in HI until the 1980s, but in the last half-century, positive genetic gains were reported for HI.

Average N fertilizer use in maize production in SSA is estimated at 17.9 kg N ha^-1^, with fertilizer use often lower on female-managed plots or within female-headed households (Jayne and Sanchez, 2021; Cairns et al., 2022). Using historical trial data between 2013 and 2021, Prasanna et al. (2022) found no significant yield gains under low N stress in this pipeline. The period used by Prasanna et al. (2022) covered the separation of the low N breeding pipeline and subsequent merger with multiple stress-tolerant maize breeding programs in ESA. The results from the present study suggest that significant genetic gains are being made within the early-maturity maize breeding pipeline in Southern Africa.

Era trials that compare the performances of older and newer cultivars in a common set of environments and management conditions have been the most popular methods used to estimate genetic gains attributable to the effects of plant breeding and selection (Rutkoski, 2019). Several studies were conducted using this approach (Duvick, 2005; Badu-Apraku et al., 2017; Masuka et al., 2017a; Masuka et al., 2017b; Badu-Apraku et al., 2021; Badu-Apraku et al., 2022; Menkir et al., 2022; Asea et al., 2023). Unlike the use of historical data from long-term trials, the genetic gains estimated using data from era trials are not confounded due to nongenetic trends raising from change in agronomic practices and increased climate variability (Piepho et al, 2014; Rutkoski, 2019; Kumar et al., 2021; Rahman et al., 2023). The results of this study suggest that the gains observed in GY and other important traits of CIMMYT’s early maturing maize hybrid breeding program were associated with genetic improvement efforts.

## Conclusions

Increased investment in hybrid maize breeding in Southern Africa and a culture of continuous improvement over the past two decades has resulted in significant genetic gains in GY across a range of stress and non-stress environments. Gains were higher than those reported by Masuka et al. (2017a); that study included only candidate hybrids up to 2010 and did not coincide with increased donor investment in stress-tolerant maize breeding in Southern Africa through various projects (e.g., DTMA, WEMA, IMAS, and STMA). Our results highlight that these investments have translated into significant genetic gains in CIMMYT-Southern African early-maturity maize breeding program. The study also underscores the importance of smallholder farmers having access to a steady stream of improved early maturing maize varieties adapted to today’s climate. One of the commercial checks included in this study (SC513) was released before the first candidate hybrid included in this study; SC513 remained a market-dominant hybrid over the past two decades. While absolute gains in farmers’ fields could be lower than on-station yield levels, by growing more recently released multiple stress-tolerant maize varieties, smallholder farmers will have greater opportunity to benefit from these gains and improve their food security, climate adaptation, and livelihoods. Even small gains in GY on-farm can have significant impact on the livelihoods of resource-constrained smallholder farmers (Hansen et al., 2019; Lunduka et al., 2019).

To realize sustainable progress in terms of genetic gains in the maize breeding pipelines especially in the stress-prone tropical environments, continuous investment is required. This will enable integration of innovative breeding tools and technologies as well as continued utilization of an expanded germplasm testing networks in the relevant target population of environments to improve selection efficiency. Genetic gains observed in on-station trials of maize breeding pipelines need to be successfully translated into gains on-farm through timely replacement of old and obsolete hybrids with new and more productive genetics that can improve the productivity of targeted farming communities.

## Data availability statement

The original contributions presented in the study are included in the article/**Supplementary Material**. Further inquiries can be directed to the corresponding author.

## Author contributions

ATa: Conceptualization, Data curation, Formal analysis, Investigation, Methodology, Project administration, Supervision, Validation, Visualization, Writing – original draft, Writing – review & editing, Resources. DW: Data curation, Formal analysis, Investigation, Methodology, Validation, Visualization, Writing – original draft, Writing – review & editing. JC: Investigation, Methodology, Validation, Visualization, Writing – review & editing. MZ-A: Investigation, Methodology, Validation, Visualization, Writing – review & editing. YB: Investigation, Methodology, Resources, Validation, Visualization, Writing – review & editing, Funding acquisition. DN: Investigation, Writing – review & editing. ATe: Investigation, Methodology, Validation, Visualization, Writing – review & editing. KT: Methodology, Resources, Software, Visualization, Writing – review & editing. MJ: Investigation, Writing – review & editing. BD: Data curation, Investigation, Resources, Writing – review & editing. EN: Investigation, Writing – review & editing. KS: Investigation, Writing – review & editing. KK: Investigation, Writing – review & editing. KM: Investigation, Writing – review & editing. NT: Investigation, Writing – review & editing. XM: Writing – review & editing. BP: Funding acquisition, Resources, Writing – review & editing.

## Supplementary material

Supplementary Figure 1 Percent grain yield (t ha^-1^) advantages of era hybrids over the mean of benchmark commercial checks under optimal, low N, managed drought and random stress environments in the era trials conducted in 2018 and 2019.

Supplementary Figure 2 Relative grain yield reduction (%) under stress environments as compared to optimal management.
