# Supplementary material for: Genetic gains in early maturing maize hybrids developed by the International Maize and Wheat Improvement Center in Southern Africa during 2000–2018
Source: Front Plant Sci. 2024 Jan 16;14:1321308. doi: 10.3389/fpls.2023.1321308 (PMC10825029; doi:10.3389/fpls.2023.1321308)
Supplement: Supplementary file 1 [file Table_1.docx]

Supplementary Table 1. Description of testing locations used to evaluate the era hybrids under optimum (OPT), low nitrogen stress (LN), managed drought stress (MD) and random stress (RS) conditions in 2018 and 2019 in Eastern and Southern Africa.

| Country | Location | Year | Manage-ment | Trial number | Latitude | longitude | Altitude (masl) | Maga-environment* | Soil Classification | Soil texture | Tmax  (^o^C) | Tmin  (^o^C) | Annual rainfall (mm) | Seaonal  rainfall  (mm) | Soil  pH |
| --- | --- | --- | --- | --- | --- | --- | --- | --- | --- | --- | --- | --- | --- | --- | --- |
| Zimbabwe | CIMMYT Harare | 2018 | OPT | 12 | -17.83 | 31.05 | 1508 | WUMA | Chromic Luvisols | Clay (heavy) | 28.6 | 6.3 | 820 | 527 | 5.6 |
| Zimbabwe | CIMMYT Harare | 2019 | OPT | 13 | -17.83 | 31.05 | 1508 | WUMA | Chromic Luvisols | Clay (heavy) | 28.6 | 6.3 | 820 | 527 | 5.6 |
| Zimbabwe | Muzarabani | 2018 | OPT | 15 | -16.37 | 30.99 | 392 | DLL | Eutric Cambisols | loamy sand | 33.6 | 8.8 | 846 | 646 | 7.0 |
| Zimbabwe | Kwekwe | 2018 | OPT | 17 | -18.82 | 29.82 | 1186 | WLMA | Haplic Lixisols | loamy sand | 31 | 5.6 | 654 | 433 | 5.9 |
| Zambia | Mpongwe | 2018 | OPT | 18 | -13.31 | 28.1 | 1162 | WUMA | orthi-xanthic Ferralsols | Clay loam | 32.1 | 6.3 | 1167 | 768 | 5.4 |
| Zambia | Lusaka-West | 2018 | OPT | 19 | -15.37 | 28.09 | 1203 | WUMA | orthi-eutric Leptosols | Clay loam | 31.7 | 8.3 | 810 | 572 | 6.4 |
| Zambia | Chongwe | 2018 | OPT | 21 | -15.31 | 28.68 | 1059 | WLMA | dystric Leptosol | sandy clay loam | 32.2 | 8.2 | 811 | 575 | 6.3 |
| Malawi | Chitala | 2018 | OPT | 31 | -13.77 | 34.47 | 502 | WLMA | Vertisols | loam sandy | 32.6 | 14.8 | 1140 | 800 | 6.6 |
| Malawi | Baka | 2018 | OPT | 33 | -9.95 | 33.92 | 487 | WLMA | Fluvisol | sandy loam | 32.8 | 17.1 | 1180 | 671 | 6.3 |
| Zimbabwe | Chinhoyi | 2018 | OPT | 37 | -17.35 | 30.21 | 1144 | WUMA | Chromic Cambisols | sandy clay loam | 31 | 6.2 | 805 | 522 | 5.8 |
| Zimbabwe | University of Zimbabwe Farm | 2018 | OPT | 38 | -17.72 | 31.02 | 1498 | WUMA | Chromic Luvisols | Clay (heavy) | 28.2 | 5.4 | 863 | 563 | 5.7 |
| Zimbabwe | Kadoma Cotton Research Station | 2018 | OPT | 39 | -18.33 | 29.92 | 1164 | WLMA | Chromic Luvisols | Clay (heavy) | 31.7 | 7 | 712 | 472 | 5.7 |
| Zambia | Nagwaza | 2018 | OPT | 40 | -12.61 | 27.87 | 1357 | WUMA | orthic-dystric Leptosols | clay loam | 31.3 | 4.3 | 1237 | 799 | 5.2 |
| Zimbabwe | Lionsden | 2018 | OPT | 41 | -17.25 | 30.02 | 1232 | WLMA | Chromic Cambisols | loamy sand | 29.5 | 6.1 | 806 | 541 | 6.0 |
| Zimbabwe | Lionsden | 2019 | OPT | 42 | -17.25 | 30.02 | 1232 | WLMA | Chromic Cambisols | loamy sand | 29.5 | 6.1 | 806 | 541 | 6.0 |
| Zimbabwe | Gwebi | 2018 | OPT | 48 | -17.68 | 30.87 | 1453 | WUMA | Chromic Luvisols | Clay (heavy) | 28.5 | 4.9 | 820 | 533 | 5.9 |
| Zimbabwe | Rattray-Arnold | 2018 | OPT | 51 | -17.67 | 31.17 | 1462 | WUMA | Hypoluvic Arenosols | sand | 28.1 | 5.3 | 892 | 589 | 5.3 |
| Zimbabwe | Glendale | 2018 | OPT | 53 | -17.36 | 31.06 | 1147 | WUMA | Rhodic Ferralsols | clay (light) | 27.8 | 3.9 | 856 | 577 | 6.0 |
| Mozambique | Lichinga | 2018 | OPT | 57 | -13.29 | 35.24 | 1359 | WUMA | Ferri-Profondic Lixisols | clay (light) | 27.8 | 9.7 | 1124 | 690 | 5.5 |
| South Africa | Cedara | 2018 | OPT | 58 | -29.55 | 30.27 | 1113 | WUMA | Rhodic Ferralsols | sandy clay loam | 25.8 | 2.9 | 820 | 369 | 5.7 |
| South Africa | Potshofstroom | 2018 | OPT | 59 | -26.68 | 27.08 | 1372 | DMA | Rhodic Lixisols | sandy clay loam | 28.6 | 0.4 | 614 | 293 | 6.0 |
| Zimbabwe | Kwekwe | 2019 | OPT | 62 | -18.82 | 29.82 | 1186 | WLMA | Haplic Lixisols | loamy sand | 31 | 5.6 | 654 | 433 | 5.9 |
| Ethiopia | Bako | 2018 | OPT | 63 | 9.1 | 37.15 | 1650 | WUMA | Endoeutric Nitisols | Clay loam | 25.5 | 13 | 1009 | 680 | 6.0 |
| Ethiopia | Ambo | 2018 | OPT | 66 | 8.95 | 38.12 | 2312 | HL | Chromic Luvisols | Clay (heavy) | 23.8 | 11.2 | 784 | 540 | 7.2 |
| Kenya | Mtwapa | 2018 | OPT | 73 | -4.35 | 39.22 | 30 | DLL | Eutric Fluvisols | loam | 32.5 | 13.3 | 485 | 276 | 7.1 |
| Kenya | Kiboko | 2018 | OPT | 75 | -2.21 | 37.73 | 975 | DMA | Calcaric Regosols | sandy clay loam | 33.8 | 15.2 | 311 | 146 | 6.5 |
| Kenya | Kirinyaga | 2018 | OPT | 76 | -0.56 | 37.32 | 999 | DMA | Haplic Lixisols | clay (light) | 33.1 | 13.8 | 752 | 352 | 7.2 |
| Kenya | Kakamega | 2018 | OPT | 78 | 0.28 | 34.76 | 1562 | WUMA | Haplic Ferralsols | clay (heavy) | 29.9 | 11 | 1896 | 683 | 5.5 |
| Zimbabwe | Gwebi | 2019 | OPT | 81 | -17.68 | 30.87 | 1453 | WUMA | Chromic Luvisols | clay (heavy) | 28.5 | 4.9 | 820 | 533 | 5.9 |
| Zimbabwe | Harare Misting | 2018 | OPT | 87 | -17.72 | 31.02 | 1498 | WUMA | Chromic Luvisols | clay (heavy) | 28.2 | 5.4 | 863 | 563 | 5.7 |
| Zimbabwe | UZ Farm | 2019 | OPT | 88 | -17.72 | 31.02 | 1498 | WUMA | Chromic Luvisols | clay (heavy) | 28.2 | 5.4 | 863 | 563 | 5.7 |
| Zimbabwe | Gweru | 2018 | OPT | 89 | -19.13 | 29.66 | 1313 | DMA | Chromic Luvisols | clay loam | 29.9 | 5.1 | 648 | 419 | 5.7 |
| Zambia | Lusaka West | 2018 | LN | 20 | -15.37 | 28.09 | 1203 | WUMA | orthi-eutric Leptosols | Clay loam | 31.7 | 8.3 | 810 | 572 | 6.4 |
| Malawi | Chitedze | 2018 | LN | 30 | -13.8 | 33.65 | 1199 | WUMA | Ferric Luvisols | sandy clay loam | 29.2 | 8.6 | 883 | 601 | 5.8 |
| Malawi | Bvumbwe | 2018 | LN | 32 | -15.62 | 35.09 | 770 | WLMA | Combisols | sandy clay loam | 30.8 | 12.6 | 985 | 608 | 5.1 |
| Zimbabwe | CIMMYT Harare | 2018 | LN | 45 | -17.73 | 31.02 | 1488 | WUMA | Chromic Luvisols | clay (heavy) | 28.1 | 5.5 | 863 | 563 | 5.8 |
| Zimbabwe | Gwebi | 2018 | LN | 50 | -17.68 | 30.87 | 1453 | WUMA | Chromic Luvisols | clay (heavy) | 28.5 | 4.9 | 820 | 533 | 5.9 |
| Zimbabwe | Rattray-Arnold | 2018 | LN | 52 | -17.67 | 31.17 | 1462 | WUMA | Hypoluvic Arenosols | sand | 28.1 | 5.3 | 892 | 589 | 5.3 |
| Mozambique | Umbuluzi | 2018 | LN | 56 | -18.39 | 35.45 | 129 | WLL | Rubi-Hypoluvic Arenosols | loamy sand | 33.9 | 14.8 | 995 | 548 | 5.6 |
| South Africa | Potchofstroom | 2018 | LN | 60 | -26.68 | 27.08 | 1372 | DMA | Rhodic Lixisols | sandy clay loam | 28.6 | 0.4 | 614 | 293 | 6 |
| South Africa | Cedara | 2018 | LN | 61 | -29.55 | 30.27 | 1113 | WUMA | Rhodic Ferralsols | sandy clay loam | 25.8 | 2.9 | 820 | 369 | 5.7 |
| Ethiopia | Bako | 2018 | LN | 64 | 9.1 | 37.15 | 1650 | WUMA | Endoeutric Nitisols | Clay loam | 25.5 | 13 | 1009 | 680 | 6 |
| Ethiopia | Ambo | 2018 | LN | 65 | 8.95 | 38.12 | 2312 | HL | Chromic Luvisols | Clay (heavy) | 23.8 | 11.2 | 784 | 540 | 7.2 |
| Kenya | Kiboko | 2018 | LN | 70 | -2.21 | 37.73 | 975 | DLL | Calcaric Regosols | sandy clay loam | 33.8 | 15.2 | 311 | 146 | 6.5 |
| Kenya | Kakamega | 2018 | LN | 71 | 0.28 | 34.76 | 1585 | WUMA | Haplic Ferralsols | clay (heavy) | 29.9 | 11 | 1896 | 683 | 5.5 |
| Zimbabwe | Rattrey Arnold | 2019 | LN | 90 | -17.73 | 31.02 | 1488 | WUMA | Chromic Luvisols | clay (heavy) | 28.1 | 5.5 | 863 | 563 | 5.8 |
| Zimbabwe | Chisumbanje | 2018A | MD | 2 | -20.76 | 32.23 | 413 | DLL | Eutric Leptosols | loam | 32 | 8.2 | 546 | 335 | 7.4 |
| Zimbabwe | Chiredzi | 2018A | MD | 4 | -21 | 31.55 | 455 | DLL | Chromic Luvisols | sandy loam | 32.4 | 8.1 | 574 | 343 | 6.9 |
| Zimbabwe | Chiredzi | 2018B | MD | 9 | -21 | 31.55 | 455 | DLL | Chromic Luvisols | sandy loam | 32.4 | 8.1 | 574 | 343 | 6.9 |
| Kenya | Kiboko | 2018 | MD | 72 | -2.21 | 37.73 | 975 | DLL | Calcaric Regosols | sandy clay loam | 33.8 | 15.2 | 311 | 146 | 6.5 |
| Kenya | Kiboko | 2018 | MD | 74 | -2.21 | 37.73 | 975 | DLL | Calcaric Regosols | sandy clay loam | 33.8 | 15.2 | 311 | 146 | 6.5 |
| Zimbabwe | Chisumbanje | 2018B | MD | 79 | -20.76 | 32.23 | 413 | DLL | Eutric Leptosols | loam | 32 | 8.2 | 546 | 335 | 7.4 |
| Zimbabwe | Chiredzi | 2019 | MD | 83 | -21 | 31.55 | 455 | DLL | Chromic Luvisols | sandy loam | 32.4 | 8.1 | 574 | 343 | 6.9 |
| Zimbabwe | Chisumbanje | 2019 | MD | 84 | -20.76 | 32.23 | 413 | DLL | Eutric Leptosols | loam | 32 | 8.2 | 546 | 335 | 7.4 |
| Zimbabwe | Makoholi | 2018 | MD | 85 | -19.83 | 30.77 | 1196 | DMA | Haplic Lixisols | loamy sand | 28.7 | 6.2 | 646 | 406 | 6 |
| Zimbabwe | Chiredzi | 2018A | RS | 1 | -21 | 31.55 | 455 | DLL | Chromic Luvisols | sandy loam | 32.4 | 8.1 | 574 | 343 | 6.9 |
| Zimbabwe | Chiredzi | 2018B | RS | 11 | -21 | 31.55 | 455 | DLL | Chromic Luvisols | sandy loam | 32.4 | 8.1 | 574 | 343 | 6.9 |
| Zimbabwe | Kadoma | 2018 | RS | 14 | -18.33 | 29.92 | 1164 | WLMA | Chromic Luvisols | clay (heavy) | 31.7 | 7 | 712 | 472 | 5.7 |
| Zimbabwe | Gweru | 2018 | RS | 16 | -19.13 | 29.66 | 1313 | DMA | Chromic Luvisols | clay loam | 29.9 | 5.1 | 648 | 419 | 5.7 |
| Zambia | Magobo | 2018 | RS | 22 | -13.63 | 32.64 | 1114 | WUMA | Chromic-haplic Alisols | Loamy sand | 31.9 | 11.2 | 1023 | 692 | 5.6 |
| Zambia | ZseedMFZM | 2018 | RS | 23 | -13.63 | 32.64 | 1114 | WUMA | Chromic-haplic Acrisols | Clay loam | 31.9 | 11.2 | 1023 | 692 | 5.6 |
| Zambia | Mumbwa (ETG) | 2018 | RS | 24 | -14.95 | 26.58 | 1180 | WUMA | Chromic-Haplic Lixisols | sandy loam | 32 | 6.1 | 911 | 637 | 5.5 |
| Zambia | Golden Valley | 2018 | RS | 25 | -18.15 | 30.23 | 1200 | WLMA | Rhodi-luvic Phaeozems | clay (heavy) | 30.9 | 5.9 | 756 | 505 | 5.9 |
| Zambia | Mt Makulu | 2018 | RS | 26 | -15.55 | 28.25 | 1228 | WUMA | Orthi-eutric Leptosols | loam clay | 31.9 | 8.9 | 803 | 563 | 6.2 |
| Malawi | Chitedze | 2018 | RS | 28 | -13.8 | 33.65 | 1199 | WUMA | Ferric Luvisols | sandy clay loam | 29.2 | 8.6 | 883 | 601 | 5.8 |
| Zimbabwe | Bindura | 2018 | RS | 35 | -17.29 | 31.32 | 1116 | WUMA | Rhodic Ferralsols | clay (light) | 30 | 4.8 | 830 | 574 | 5.7 |
| Zambia | Kapiliyomba | 2018 | RS | 44 | -12.42 | 27.8 | 1286 | WUMA | Chromi-haplic Luvisols | clay loam | 31.8 | 4.8 | 1216 | 788 | 5.3 |
| Ethiopia | Melkassa | 2018 | RS | 67 | 8.4 | 39.35 | 1570 | DMA | Andosol | sandy loam | 28.5 | 14 | 750 | 510 | 7.5 |
