# Supplementary material for: Genetic gains in early maturing maize hybrids developed by the International Maize and Wheat Improvement Center in Southern Africa during 2000–2018
Source: Front Plant Sci. 2024 Jan 16;14:1321308. doi: 10.3389/fpls.2023.1321308 (PMC10825029; doi:10.3389/fpls.2023.1321308)
Supplement: Supplementary file 2 [file Table_2.docx]

Supplementary Table 2. Means and genetic parameters for grain yield and other agronomic traits of early maturing maize hybrids evaluated across 68 stress and non-stress environments in 2018 and 2019 in Eastern and Southern Africa.

| Mean/  variance | GY^+^ | AD | SD | ASI | PH | EH | EPO | RL | SL | EPP | TEX | BHC | ER | HI | PA | EA | GLS | CR | TL B | SEN |
| --- | --- | --- | --- | --- | --- | --- | --- | --- | --- | --- | --- | --- | --- | --- | --- | --- | --- | --- | --- | --- |
| Heritability | 0.98 | 0.99 | 0.96 | 0.93 | 0.99 | 0.99 | 0.98 | 0.90 | 0.86 | 0.91 | 0.98 | 0.90 | 0.91 | 0.78 | 0.68 | 0.94 | 0.91 | 0.79 | 0.82 | 0.94 |
| 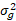 | 0.37** | 3.28** | 3.44** | 0.16** | 114.82** | 64.37** | 0** | 3.23** | 1.21** | 0** | 0.15** | 2.46** | 1.91** | 14.95** | 0.05** | 0.02** | 0.08** | 0.02** | 0.02** | 0.03** |
| 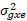 | 0.27** | 1.11** | 1.36** | 0.25** | 27.08** | 21.28** | 0** | 9.54** | 3.89** | 0** | 0.05** | 9.33** | 7.35** | 7.05** | 0.08** | 0.05** | 0.43** | 0.17** | 0.14** | 0.07** |
| 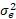 | 5.98** | 65.11** | 66.43** | 1.32** | 1112 ** | 447** | 0** | 37.63** | 21.91** | 0.02** | 0.28** | 29.71** | 31.48** | 73.32** | 0.15** | 0.22** | 1.48** | 1.8** | 1.54** | 3.06** |
| $\sigma_{resid}^{2}$ | 0.95 | 3.10 | 2.67 | 2.24 | 201.83 | 148.15 | 0.00 | 53.76 | 35.53 | 0.03 | 0.55 | 33.77 | 24.71 | 40.44 | 0.23 | 0.17 | 0.53 | 0.44 | 0.53 | 0.27 |
| Mean | 5.50 | 67.30 | 68.83 | 1.53 | 212.00 | 105.00 | 0.49 | 5.44 | 3.99 | 0.99 | 2.69 | 4.26 | 6.09 | 41.82 | 2.66 | 3.01 | 3.77 | 4.01 | 3.61 | 4.93 |
| Minimum | 4.00 | 62.50 | 63.22 | 0.72 | 170.00 | 83.00 | 0.44 | 3.10 | 1.78 | 0.93 | 1.81 | 1.90 | 3.32 | 29.03 | 1.91 | 2.67 | 2.68 | 3.36 | 2.80 | 4.50 |
| Maximum | 6.70 | 71.50 | 74.35 | 2.85 | 230.00 | 122.00 | 0.54 | 11.77 | 7.63 | 1.12 | 3.64 | 9.21 | 13.17 | 54.80 | 3.40 | 3.44 | 5.22 | 4.84 | 4.66 | 5.54 |
| LSD | 1.90 | 3.50 | 6.43 | 2.93 | 28.00 | 24.00 | 0.10 | 14.37 | 11.68 | 0.31 | 1.45 | 11.39 | 9.74 | 5.70 | 0.45 | 0.80 | 1.42 | 1.30 | 1.42 | 1.03 |
| CV | 17.6 | 2.6 | 2.6 | 97.9 | 7.0 | 12.0 | 10.2 | 134.7 | 149.3 | 15.9 | 27.6 | 136.6 | 81.6 | 15.2 | 18.0 | 13.6 | 30.4 | 26.5 | 25.9 | 10.6 |
| #Locs | 68 | 68 | 68 | 46 | 66 | 64 | 56 | 35 | 32 | 31 | 31 | 28 | 29 | 5 | 6 | 42 | 9 | 11 | 13 | 8 |

^+^GY, Grain yield (t ha^-1^); AD, Days to anthesis (d); SD, Days to silking (d); ASI, Anthesis-silking interval (d); PH, Plant height (cm); EH, Ear height (cm); EPO, Ear position; RL, Root lodging (%); SL, Stalk lodging (%); EPP, Number of ears per plant; TEX, Kernel texture (1-5); BHC, Bad husk cover (%); ER, Ear rot (%); HI, Harvest index (%); PA, Plant aspect (1-5); EA, Ear aspect (1-5); GLS, Grey leaf spot (1-9); CR, Common rust (1-9); TLB, Turcicum leaf blight (1-9); SEN, Leaf senescence (1 – 10 scale).
