# Supplementary material for: Genetic gains in early maturing maize hybrids developed by the International Maize and Wheat Improvement Center in Southern Africa during 2000–2018
Source: Front Plant Sci. 2024 Jan 16;14:1321308. doi: 10.3389/fpls.2023.1321308 (PMC10825029; doi:10.3389/fpls.2023.1321308)
Supplement: Supplementary file 3 [file Table_3.docx]

Supplementary Table 3. Mean grain yield and other traits of 72 era hybrids and three commercial checks evaluated under across optimal and stress environments in Eastern and Southern Africa from 2018 to 2019

| Entry | Hybrid* | Year of  release | Grain yield (t/ha) | | | | AD^+^ | SD | ASI | PH | EH | RL | SL | EPP | ER | GLS | CR | TLB | Tex | HI |
| --- | --- | --- | --- | --- | --- | --- | --- | --- | --- | --- | --- | --- | --- | --- | --- | --- | --- | --- | --- | --- |
|  |  |  | Optimal | Random stress | Managed drought | Low N stress | d | d | d | cm | cm | % | % | # | # | 1-9 | 1-9 | 1-9 | 1-5 | % |
| 1 | SC513 | 1999 | 6.2 | 3.1 | 1.8 | 2.5 | 65 | 67 | 1.29 | 238 | 121 | 13.69 | 7.01 | 1.00 | 11.4 | 2.64 | 2.44 | 3.67 | 3.3 | 54.8 |
| 2 | SC403 | 1998 | 6.3 | 3.1 | 1.8 | 2.4 | 62 | 64 | 1.91 | 235 | 111 | 4.12 | 3.00 | 1.03 | 10.8 | 4.23 | 2.55 | 3.82 | 2.6 | 49.2 |
| 3 | PAN413 | 1998 | 5.7 | 3.0 | 1.7 | 2.4 | 66 | 68 | 2.12 | 208 | 104 | 9.66 | 6.76 | 1.04 | 14.3 | 4.25 | 2.61 | 4.01 | 3.9 | 43.2 |
| 4 | CZH99015 | 2000 | 6.5 | 4.0 | 2.4 | 3.1 | 65 | 66 | 1.63 | 222 | 107 | 1.53 | 4.31 | 1.02 | 4.7 | 4.09 | 2.73 | 3.53 | 1.8 | 44.5 |
| 5 | CZH99014 | 2000 | 6.4 | 3.9 | 2.3 | 3.1 | 66 | 67 | 1.33 | 220 | 114 | 4.59 | 6.55 | 1.03 | 4.4 | 3.49 | 2.64 | 3.38 | 2.0 | 44.5 |
| 6 | CZH99019 | 2000 | 7.0 | 3.7 | 2.3 | 2.8 | 67 | 69 | 1.31 | 247 | 130 | 11.64 | 10.25 | 1.07 | 4.8 | 4.05 | 2.25 | 3.33 | 1.9 | 48.1 |
| 7 | CZH00011 | 2001 | 5.8 | 3.3 | 2.3 | 2.8 | 62 | 63 | 0.99 | 227 | 113 | 7.38 | 7.70 | 1.03 | 7.4 | 4.79 | 2.67 | 4.78 | 2.1 | 41.1 |
| 8 | CZH00013 | 2001 | 6.6 | 3.4 | 2.4 | 2.6 | 64 | 65 | 0.84 | 247 | 124 | 9.65 | 5.94 | 1.06 | 5.5 | 4.91 | 2.78 | 4.68 | 2.3 | 35.7 |
| 9 | CZH00020 | 2001 | 7.5 | 4.0 | 2.2 | 2.9 | 69 | 70 | 1.01 | 241 | 133 | 5.71 | 8.36 | 1.07 | 2.9 | 3.97 | 1.95 | 4.03 | 3.0 | 41.4 |
| 10 | CZH01006 | 2002 | 6.6 | 3.8 | 2.3 | 2.8 | 65 | 66 | 0.93 | 245 | 127 | 5.63 | 5.17 | 1.13 | 6.2 | 4.58 | 2.67 | 4.03 | 2.6 | 40.1 |
| 11 | CZH01008 | 2002 | 7.8 | 4.3 | 2.1 | 2.8 | 70 | 70 | 0.35 | 252 | 135 | 7.14 | 6.48 | 1.25 | 7.3 | 2.85 | 2.31 | 3.45 | 3.1 | 41.8 |
| 12 | CZH02008 | 2003 | 6.7 | 3.8 | 2.2 | 2.9 | 61 | 64 | 2.69 | 232 | 112 | 8.31 | 6.41 | 1.04 | 6.8 | 5.37 | 2.12 | 5.24 | 2.3 | 45.4 |
| 13 | CZH02006 | 2003 | 7.5 | 3.8 | 2.2 | 3.1 | 64 | 66 | 1.34 | 230 | 109 | 3.00 | 5.25 | 1.24 | 6.0 | 4.67 | 2.43 | 4.34 | 3.0 | 41.7 |
| 14 | CZH02004 | 2003 | 6.9 | 4.3 | 2.6 | 3.0 | 64 | 66 | 1.37 | 214 | 107 | 1.90 | 3.79 | 1.05 | 6.4 | 4.65 | 2.49 | 4.51 | 2.8 | 46.5 |
| 15 | CZH03005 | 2004 | 7.7 | 4.1 | 2.3 | 3.2 | 66 | 68 | 1.42 | 247 | 128 | 5.22 | 6.02 | 1.20 | 4.6 | 4.12 | 2.52 | 4.12 | 2.7 | 41.6 |
| 16 | CZH03006 | 2004 | 7.6 | 4.0 | 2.5 | 3.3 | 65 | 66 | 0.64 | 231 | 120 | 2.47 | 2.33 | 1.05 | 6.3 | 3.05 | 2.30 | 2.75 | 3.0 | 47.1 |
| 17 | CZH03002 | 2004 | 7.5 | 4.3 | 2.8 | 3.1 | 68 | 68 | 0.09 | 243 | 126 | 1.63 | 2.70 | 1.05 | 5.6 | 2.80 | 2.55 | 3.28 | 2.8 | 38.3 |
| 18 | CZH04001 | 2005 | 7.9 | 4.6 | 2.3 | 2.7 | 69 | 70 | 0.63 | 244 | 125 | 4.27 | 3.96 | 1.04 | 3.3 | 4.24 | 2.33 | 3.40 | 2.3 | 35.4 |
| 19 | CZH04005 | 2005 | 7.1 | 4.1 | 2.5 | 3.5 | 65 | 66 | 1.13 | 229 | 125 | 3.14 | 5.47 | 1.08 | 4.6 | 4.51 | 2.20 | 3.86 | 2.5 | 39.0 |
| 20 | CZH04007 | 2005 | 7.9 | 4.7 | 2.5 | 3.6 | 67 | 68 | 0.29 | 232 | 118 | 1.19 | 2.38 | 1.11 | 6.0 | 2.27 | 1.81 | 3.22 | 2.9 | 38.2 |
| 21 | CZH0524 | 2006 | 7.0 | 3.8 | 2.8 | 3.1 | 63 | 64 | 1.08 | 234 | 110 | 8.38 | 1.33 | 1.04 | 7.2 | 4.13 | 2.17 | 3.19 | 2.5 | 40.4 |
| 22 | CZH0528 | 2006 | 6.7 | 3.7 | 2.4 | 3.1 | 64 | 66 | 1.32 | 221 | 106 | 2.77 | 3.88 | 1.03 | 5.0 | 4.08 | 2.23 | 4.12 | 1.8 | 41.9 |
| 23 | CZH0527 | 2006 | 7.2 | 4.1 | 2.3 | 3.3 | 65 | 67 | 1.81 | 233 | 112 | 3.54 | 3.49 | 1.02 | 8.5 | 4.06 | 2.17 | 3.87 | 2.8 | 42.2 |
| 24 | CZH0526 | 2006 | 7.6 | 3.7 | 2.4 | 3.0 | 66 | 67 | 1.75 | 238 | 121 | 3.57 | 5.89 | 1.00 | 6.0 | 3.24 | 2.29 | 3.40 | 2.4 | 40.9 |
| 25 | CZH0629 | 2007 | 7.7 | 4.2 | 2.4 | 3.5 | 65 | 66 | 0.89 | 228 | 121 | 3.64 | 2.03 | 1.17 | 6.8 | 2.43 | 2.01 | 3.24 | 3.3 | 41.1 |
| 26 | CZH0623 | 2007 | 8.0 | 4.7 | 2.5 | 3.7 | 67 | 68 | 0.73 | 236 | 119 | 1.61 | 3.03 | 1.15 | 6.2 | 3.20 | 2.56 | 3.38 | 2.8 | 37.6 |
| 27 | CZH0615 | 2007 | 7.7 | 4.6 | 2.3 | 3.6 | 65 | 66 | 1.43 | 231 | 116 | 2.04 | 4.78 | 1.09 | 6.2 | 3.34 | 2.28 | 3.53 | 2.5 | 39.4 |
| 28 | CZH0735 | 2008 | 6.3 | 3.5 | 2.2 | 3.0 | 61 | 63 | 1.45 | 211 | 108 | 8.81 | 8.32 | 1.02 | 5.0 | 4.37 | 2.29 | 4.14 | 2.3 | 49.2 |
| 29 | CZH0728 | 2008 | 7.5 | 4.3 | 2.2 | 3.0 | 69 | 70 | 0.56 | 248 | 137 | 4.40 | 4.55 | 1.16 | 4.4 | 4.45 | 2.85 | 3.83 | 2.6 | 39.8 |
| 30 | CZH0733 | 2008 | 7.2 | 4.1 | 2.2 | 3.4 | 64 | 66 | 1.85 | 228 | 112 | 2.98 | 4.46 | 1.04 | 8.2 | 3.27 | 2.47 | 3.48 | 3.1 | 42.6 |
| 31 | CZH089 | 2009 | 6.8 | 4.1 | 2.4 | 3.4 | 64 | 66 | 1.78 | 209 | 101 | 1.60 | 2.49 | 1.01 | 6.6 | 3.46 | 2.27 | 3.23 | 3.3 | 45.1 |
| 32 | CZH0836 | 2009 | 8.5 | 4.4 | 2.3 | 3.2 | 68 | 69 | 1.33 | 252 | 133 | 1.65 | 3.78 | 1.04 | 5.5 | 2.26 | 2.06 | 2.92 | 3.2 | 41.2 |
| 33 | CZH088 | 2009 | 6.3 | 3.6 | 2.0 | 3.1 | 60 | 62 | 2.38 | 185 | 90 | 3.29 | 3.40 | 1.00 | 10.3 | 2.59 | 2.33 | 4.55 | 1.6 | 47.3 |
| 34 | CZH0946 | 2010 | 6.5 | 3.6 | 2.3 | 3.3 | 60 | 60 | 0.05 | 201 | 100 | 1.14 | 3.58 | 1.05 | 7.3 | 3.98 | 2.00 | 3.98 | 1.7 | 40.8 |
| 35 | CZH0932 | 2010 | 7.7 | 4.3 | 2.5 | 3.6 | 66 | 67 | 0.86 | 226 | 119 | 5.20 | 2.67 | 1.11 | 6.0 | 3.66 | 2.65 | 3.44 | 2.2 | 37.7 |
| 36 | CZH0935 | 2010 | 8.2 | 4.2 | 2.8 | 3.7 | 65 | 67 | 1.52 | 232 | 114 | 1.75 | 3.02 | 1.07 | 6.7 | 2.84 | 2.61 | 3.42 | 2.1 | 44.3 |
| 37 | CZH0928 | 2010 | 7.9 | 4.5 | 2.9 | 3.8 | 65 | 66 | 0.87 | 229 | 109 | 2.88 | 2.57 | 1.07 | 5.9 | 2.98 | 2.58 | 3.29 | 2.4 | 36.1 |
| 38 | CZH1033 | 2011 | 7.9 | 4.4 | 2.6 | 3.6 | 66 | 66 | 0.55 | 220 | 116 | 3.90 | 4.73 | 1.10 | 2.4 | 2.10 | 2.39 | 2.64 | 2.2 | 40.2 |
| 39 | CZH1036 | 2011 | 7.9 | 4.4 | 2.9 | 3.8 | 65 | 66 | 1.21 | 230 | 117 | 2.60 | 1.50 | 1.10 | 5.7 | 2.93 | 2.41 | 3.43 | 3.1 | 39.9 |
| 40 | CZH1032 | 2011 | 7.5 | 4.1 | 2.5 | 3.4 | 66 | 67 | 1.05 | 218 | 107 | 1.79 | 3.65 | 1.19 | 5.7 | 3.12 | 2.83 | 3.05 | 2.6 | 35.3 |
| 41 | CZH1122 | 2012 | 7.0 | 4.0 | 2.4 | 3.4 | 62 | 64 | 1.63 | 209 | 99 | 2.12 | 3.03 | 1.01 | 7.6 | 4.04 | 2.64 | 4.37 | 2.8 | 47.0 |
| 42 | CZH1123 | 2012 | 7.0 | 3.9 | 2.6 | 3.4 | 62 | 64 | 1.91 | 216 | 103 | 4.13 | 4.29 | 1.02 | 4.5 | 4.73 | 2.20 | 4.52 | 2.0 | 44.7 |
| 43 | CZH1134 | 2012 | 7.3 | 3.9 | 2.6 | 3.4 | 63 | 65 | 2.04 | 226 | 103 | 3.33 | 2.27 | 1.02 | 9.3 | 3.39 | 2.43 | 3.56 | 2.8 | 45.6 |
| 44 | CZH1258 | 2013 | 8.3 | 4.3 | 2.9 | 3.4 | 64 | 66 | 1.45 | 235 | 120 | 1.63 | 1.79 | 1.08 | 5.2 | 3.30 | 2.06 | 3.73 | 3.4 | 45.7 |
| 45 | CZH1261 | 2013 | 8.1 | 4.4 | 2.9 | 3.6 | 64 | 65 | 0.69 | 235 | 122 | 1.86 | 3.01 | 1.11 | 5.8 | 3.27 | 2.00 | 3.47 | 3.4 | 41.2 |
| 46 | CZH1243 | 2013 | 8.0 | 4.5 | 2.7 | 3.9 | 66 | 67 | 0.87 | 230 | 112 | 1.73 | 2.59 | 1.10 | 4.8 | 3.09 | 2.72 | 3.12 | 2.1 | 39.2 |
| 47 | CZH1270 | 2013 | 8.3 | 4.7 | 2.7 | 3.7 | 68 | 69 | 0.85 | 235 | 122 | 2.39 | 1.95 | 1.17 | 3.6 | 2.33 | 2.61 | 2.77 | 2.2 | 29.0 |
| 48 | CZH132117 | 2014 | 8.6 | 4.5 | 2.8 | 4.2 | 63 | 65 | 1.53 | 243 | 118 | 6.95 | 2.99 | 1.01 | 5.3 | 3.98 | 2.46 | 3.76 | 3.6 | 47.3 |
| 49 | CZH132119 | 2014 | 8.0 | 4.3 | 3.1 | 3.8 | 63 | 64 | 1.07 | 241 | 110 | 2.24 | 1.12 | 1.01 | 6.7 | 2.65 | 2.91 | 3.13 | 2.9 | 37.7 |
| 50 | CZH132118 | 2014 | 8.2 | 4.6 | 3.4 | 3.8 | 63 | 65 | 2.17 | 236 | 113 | 3.85 | 3.74 | 1.03 | 5.0 | 2.79 | 2.24 | 3.26 | 3.6 | 52.8 |
| 51 | CZH132169 | 2014 | 8.3 | 4.7 | 3.0 | 3.8 | 64 | 66 | 2.01 | 232 | 104 | 2.35 | 1.98 | 1.07 | 6.7 | 3.15 | 2.46 | 3.31 | 2.8 | 46.6 |
| 52 | CZH132163 | 2014 | 8.2 | 4.9 | 2.8 | 3.8 | 66 | 67 | 1.25 | 234 | 117 | 7.11 | 5.70 | 1.24 | 4.1 | 3.72 | 2.62 | 3.27 | 2.7 | 39.4 |
| 53 | CZH142056 | 2015 | 8.8 | 5.1 | 2.7 | 3.8 | 65 | 66 | 1.50 | 240 | 117 | 2.50 | 2.21 | 1.08 | 6.9 | 3.34 | 1.99 | 3.50 | 2.8 | 42.5 |
| 54 | CZH142020 | 2015 | 8.8 | 4.5 | 2.9 | 3.6 | 67 | 68 | 0.96 | 242 | 130 | 2.63 | 6.94 | 1.08 | 4.4 | 3.76 | 2.27 | 3.26 | 3.4 | 43.7 |
| 55 | CZH142055 | 2015 | 8.2 | 4.5 | 2.7 | 3.6 | 65 | 66 | 1.34 | 235 | 123 | 1.33 | 3.87 | 1.09 | 4.7 | 3.15 | 2.08 | 2.83 | 3.1 | 44.7 |
| 56 | CZH142060 | 2015 | 8.7 | 4.7 | 2.8 | 3.7 | 67 | 68 | 1.20 | 241 | 130 | 3.34 | 4.22 | 1.08 | 5.3 | 3.82 | 2.35 | 3.45 | 3.0 | 41.3 |
| 57 | CZH15002 | 2016 | 8.5 | 4.8 | 3.1 | 4.1 | 64 | 67 | 3.20 | 234 | 110 | 3.91 | 5.41 | 1.04 | 5.0 | 4.08 | 2.24 | 3.92 | 3.6 | 51.0 |
| 58 | CZH15188 | 2016 | 8.6 | 4.9 | 2.9 | 4.0 | 66 | 68 | 0.92 | 233 | 121 | 3.04 | 3.33 | 1.10 | 4.4 | 4.09 | 2.67 | 3.51 | 2.6 | 42.9 |
| 59 | CZH15017 | 2016 | 8.4 | 4.8 | 3.2 | 3.8 | 64 | 66 | 1.66 | 236 | 111 | 2.17 | 4.86 | 1.13 | 5.0 | 3.73 | 3.12 | 3.78 | 2.6 | 40.3 |
| 60 | CZH15183 | 2016 | 8.4 | 4.4 | 3.1 | 3.8 | 66 | 66 | 0.70 | 247 | 117 | 0.96 | 3.50 | 1.01 | 6.3 | 2.53 | 2.35 | 3.09 | 3.0 | 37.7 |
| 61 | CZH15013 | 2016 | 8.4 | 4.6 | 3.2 | 3.8 | 63 | 63 | 0.54 | 230 | 105 | 1.47 | 1.53 | 1.03 | 7.4 | 2.18 | 2.64 | 2.93 | 3.4 | 39.7 |
| 62 | CZH15185 | 2016 | 8.3 | 5.0 | 2.9 | 4.0 | 67 | 69 | 1.46 | 249 | 133 | 4.81 | 4.09 | 1.09 | 6.4 | 4.50 | 2.35 | 4.24 | 3.0 | 35.5 |
| 63 | CZH15575 | 2017 | 8.9 | 5.2 | 3.3 | 4.0 | 65 | 67 | 1.55 | 245 | 121 | 1.80 | 3.10 | 1.16 | 5.4 | 3.59 | 2.47 | 3.23 | 3.3 | 41.9 |
| 64 | CZH15212 | 2017 | 8.3 | 4.3 | 2.8 | 3.8 | 64 | 65 | 0.99 | 233 | 119 | 5.60 | 3.85 | 1.09 | 6.9 | 3.10 | 1.79 | 3.11 | 3.5 | 38.7 |
| 65 | CZH15467 | 2017 | 8.7 | 5.0 | 3.0 | 3.9 | 65 | 66 | 0.85 | 231 | 114 | 1.30 | 3.23 | 1.16 | 4.8 | 3.45 | 2.29 | 2.87 | 2.9 | 37.1 |
| 66 | CZH15572 | 2017 | 8.6 | 4.8 | 3.0 | 4.1 | 66 | 68 | 1.86 | 247 | 123 | 2.71 | 1.49 | 1.08 | 5.1 | 4.28 | 2.65 | 4.20 | 3.3 | 36.8 |
| 67 | CZH15603 | 2017 | 8.8 | 5.0 | 3.2 | 4.1 | 65 | 66 | 0.31 | 240 | 130 | 3.75 | 6.57 | 1.30 | 4.0 | 4.31 | 2.44 | 3.93 | 2.6 | 45.6 |
| 68 | CZH15343 | 2017 | 8.9 | 4.7 | 2.8 | 3.4 | 68 | 70 | 1.20 | 255 | 131 | 4.13 | 4.60 | 1.26 | 5.1 | 3.90 | 2.45 | 3.43 | 3.0 | 38.5 |
| 69 | CZH16333 | 2018 | 9.1 | 5.0 | 2.9 | 3.8 | 66 | 67 | 1.16 | 235 | 125 | 1.26 | 2.59 | 1.06 | 4.0 | 2.98 | 2.09 | 3.30 | 2.9 | 38.8 |
| 70 | CZH16334 | 2018 | 8.9 | 4.8 | 2.7 | 3.9 | 66 | 67 | 1.05 | 247 | 132 | 1.23 | 3.70 | 1.03 | 3.8 | 2.38 | 2.19 | 2.94 | 3.1 | 39.1 |
| 71 | CZH16389 | 2018 | 9.0 | 4.8 | 2.8 | 3.9 | 66 | 66 | 0.19 | 248 | 130 | 2.56 | 1.93 | 1.16 | 6.2 | 2.03 | 1.96 | 2.87 | 3.4 | 38.3 |
| 72 | CZH16335 | 2018 | 9.3 | 5.1 | 2.9 | 3.8 | 68 | 69 | 1.11 | 241 | 119 | 2.78 | 4.12 | 1.09 | 4.1 | 2.72 | 1.92 | 2.92 | 2.8 | 40.6 |
| 73 | CZH16340 | 2018 | 9.6 | 5.2 | 3.4 | 4.2 | 66 | 66 | -0.16 | 257 | 135 | 3.42 | 2.74 | 1.23 | 6.2 | 3.40 | 2.03 | 3.57 | 3.3 | 40.3 |
| 74 | CZH16374 | 2018 | 8.5 | 4.7 | 2.9 | 4.0 | 66 | 67 | 0.80 | 224 | 111 | 1.81 | 1.23 | 1.18 | 7.5 | 2.06 | 1.71 | 2.31 | 2.5 | 39.4 |
| 75 | CZH16048 | 2018 | 8.6 | 5.0 | 3.0 | 4.0 | 66 | 68 | 1.60 | 239 | 124 | 6.42 | 7.90 | 1.10 | 6.0 | 3.27 | 2.30 | 2.95 | 2.9 | 45.1 |
| Heritability | | | 0.97 | 0.89 | 0.84 | 0.88 | 1.0 | 1.0 | 0.9 | 1.0 | 1.0 | 0.77 | 0.63 | 0.93 | 0.36 | 0.82 | 0.13 | 0.71 | 0.98 | 0.8 |
| Environmental (E) variance | | | 0.77 | 0.24 | 0.11 | 0.18 | 4.2 | 3.6 | 0.3 | 161.5 | 97.0 | 5.17 | 2.26 | 0.00 | 1.87 | 0.51 | 0.01 | 0.22 | 0.25 | 14.9 |
| Genetic (G) variance | | | 0.29 | 0.06 | 0.11 | 0.08 | 0.6 | 0.6 | 0.2 | 23.7 | 19.0 | 10.11 | 6.77 | 0.00 | 2.24 | 0.66 | 0.34 | 0.35 | 0.03 | 7.0 |
| G × E variance | | | 1.86 | 0.57 | 0.53 | 0.52 | 31.9 | 38.3 | 0.9 | 764.2 | 224.0 | 8.49 | 39.84 | 0.01 | 1.58 | 1.37 | 2.01 | 0.75 | 0.14 | 73.3 |
| Residual variance | | | 1.24 | 0.63 | 0.27 | 0.62 | 2.1 | 2.2 | 1.70 | 178 | 145 | 38.4 | 36.7 | 0.02 | 21.1 | 1.11 | 0.60 | 0.76 | 0.2 | 40.4 |
| Mean | | | 7.77 | 4.3 | 2.6 | 3.46 | 65.1 | 66.4 | 1.22 | 233 | 117 | 3.8 | 4.1 | 1.09 | 6.0 | 3.51 | 2.37 | 3.55 | 2.8 | 41.8 |
| LSD (0.05) | | | 0.42 | 0.5 | 0.4 | 0.45 | 0.66 | 0.68 | 0.54 | 5 | 4 | 3.5 | 3.4 | 0.05 | 2.5 | 0.95 | 0.73 | 0.84 | 0.2 | 5.7 |
| CV (%) | | | 14 | 19 | 20 | 23 | 2 | 2 | 107 | 6 | 10 | 164 | 149 | 12 | 77 | 30 | 33 | 25 | 14 | 15 |
| nrep | | | 3 | 3 | 3 | 3 | 3 | 3 | 3 | 3 | 3 | 3 | 3 | 3 | 3.0 | 3 | 3 | 3 | 3.0 | 3 |
| nLoc | | | 32 | 9 | 9 | 12 | 25 | 24 | 21 | 31 | 31 | 15 | 14 | 24 | 20 | 9 | 8 | 7 | 14 | 5 |

*Hybrids SC513, SC403 and PAN413 are commercials checks

^+^AD, Days to anthesis (d); SD, Days to silking (d); Anthesis-silking interval (d); PH, Plant height (cm); EH, Ear height (cm); RL, Root lodging (%); SL, Stalk lodging (%); EPP, Ears per plant; Tex, Kernel texture (1-5); BHC, Bad husk cover (%); ER, Ear rot (%); HI, Harvest index (%); PA, Plant aspect (1-5); EA, Ear aspect (1-5); GLS, Grey leaf spot (1-9); CR, Common rust (1-9); TLB, Turcicum leaf blight (1-9).
