# Supplementary material for: Genetic gains in early maturing maize hybrids developed by the International Maize and Wheat Improvement Center in Southern Africa during 2000–2018
Source: Front Plant Sci. 2024 Jan 16;14:1321308. doi: 10.3389/fpls.2023.1321308 (PMC10825029; doi:10.3389/fpls.2023.1321308)
Supplement: Supplementary file 4 [file Table_4.docx]

Supplementary Table 4. Phenotypic (above diagonal) and genetic (below diagonal) correlation coefficients among selected stress and non-stress environments for grain yield of early maturing maize hybrids evaluated in Eastern and Southern Africa in 2018 and 2019

| S/N | Location | Country | Management | Low Nitrogen stress locations | | | | | | | Managed drought | | | | Optimum management locations | | | | | | | Random stress locations | | | | | | |
| --- | --- | --- | --- | --- | --- | --- | --- | --- | --- | --- | --- | --- | --- | --- | --- | --- | --- | --- | --- | --- | --- | --- | --- | --- | --- | --- | --- | --- |
|  |  |  |  | 1 | 2 | 3 | 4 | 5 | 6 | 7 | 8 | 9 | 10 | 11 | 12 | 13 | 14 | 15 | 16 | 17 | 18 | 19 | 20 | 21 | 22 | 23 | 24 | 25 |
| 1 | CIMMYT-Harare Station | Zimbabwe | Low Nitrogen |  | 0.55 | 0.55 | 0.46 | 0.34 | 0.31 | 0.37 | 0.05 | 0.64 | 0.34 | 0.38 | 0.48 | 0.55 | 0.56 | 0.32 | 0.42 | 0.30 | 0.30 | 0.93 | 0.73 | 0.61 | 0.69 | 0.81 | 0.65 | 0.41 |
| 2 | Gwebi | Zimbabwe | Low Nitrogen | 0.89 |  | 0.62 | 0.61 | 0.46 | 0.38 | 0.64 | 0.05 | 0.89 | 0.40 | 0.49 | 0.50 | 0.52 | 0.62 | 0.68 | 0.46 | 0.42 | 0.33 | 0.94 | 0.76 | 0.83 | 0.66 | 0.56 | 0.70 | 0.51 |
| 3 | Rattray-Arnold Research Station | Zimbabwe | Low Nitrogen | 1.00 | 1.00 |  | 0.66 | 0.32 | 0.24 | 0.39 | 0.19 | 0.88 | 0.22 | 0.52 | 0.70 | 0.59 | 0.64 | 0.73 | 0.74 | 0.34 | 0.35 | 1.00 | 0.65 | 0.86 | 0.71 | 0.74 | 0.75 | 0.44 |
| 4 | Potchofstroom | South Africa | Low Nitrogen | 0.92 | 1.00 | 1.00 |  | 0.42 | 0.18 | 0.59 | 0.11 | 0.93 | 0.26 | 0.38 | 0.75 | 0.71 | 0.88 | 0.75 | 0.82 | 0.37 | 0.40 | 0.90 | 0.95 | 0.95 | 0.75 | 0.90 | 0.84 | 0.55 |
| 5 | Bako | Ethiopia | Low Nitrogen | 0.66 | 0.76 | 0.63 | 0.85 |  | 0.36 | 0.31 | 0.15 | 0.67 | 0.28 | 0.27 | 0.40 | 0.52 | 0.45 | 0.67 | 0.62 | 0.63 | 0.39 | 0.62 | 0.57 | 0.65 | 0.46 | 0.68 | 0.63 | 0.40 |
| 6 | Kiboko | Kenya | Low Nitrogen | 0.60 | 0.62 | 0.48 | 0.37 | 0.72 |  | 0.19 | -0.02 | 0.51 | 0.34 | 0.28 | 0.29 | 0.34 | 0.24 | 0.31 | 0.40 | 0.46 | 0.25 | 0.36 | 0.54 | 0.38 | 0.37 | 0.41 | 0.67 | 0.55 |
| 7 | Kakamega | Kenya | Low Nitrogen | 0.66 | 0.98 | 0.72 | 1.00 | 0.58 | 0.34 |  | 0.16 | 0.95 | 0.24 | 0.42 | 0.73 | 0.60 | 0.87 | 0.84 | 0.67 | 0.90 | 0.49 | 0.87 | 0.81 | 0.77 | 0.66 | 0.75 | 0.59 | 0.93 |
| 8 | Chisumbanje | Zimbabwe | Managed drought | 0.03 | 0.04 | 0.12 | 0.07 | 0.10 | -0.01 | 0.11 |  | 0.17 | 0.12 | 0.17 | 0.34 | 0.50 | 0.41 | 0.40 | 0.26 | 0.61 | 0.57 | 0.15 | 0.22 | 0.30 | 0.34 | 0.13 | 0.23 | 0.29 |
| 9 | Chiredzi | Zimbabwe | Managed drought | 0.42 | 0.67 | 0.55 | 0.57 | 0.42 | 0.32 | 0.65 | 0.22 |  | 0.27 | 0.49 | 0.51 | 0.52 | 0.60 | 0.55 | 0.54 | 0.47 | 0.54 | 0.50 | 0.52 | 0.45 | 0.52 | 0.43 | 0.59 | 0.59 |
| 10 | Kiboko | Kenya | Managed drought | 0.58 | 0.58 | 0.38 | 0.46 | 0.50 | 0.58 | 0.38 | 0.17 | 0.37 |  | 0.38 | 0.13 | 0.14 | 0.33 | 0.17 | 0.30 | 0.56 | 0.36 | 0.52 | 0.29 | 0.23 | 0.59 | 0.20 | 0.42 | 0.66 |
| 11 | Makoholi | Zimbabwe | Managed drought | 0.64 | 0.72 | 0.91 | 0.67 | 0.48 | 0.48 | 0.67 | 0.23 | 0.68 | 0.58 |  | 0.33 | 0.43 | 0.50 | 0.43 | 0.32 | 0.66 | 0.59 | 0.69 | 0.51 | 0.49 | 0.60 | 0.05 | 0.62 | 0.66 |
| 12 | CIMMYT-Harare Station | Zimbabwe | Optimum | 0.32 | 0.39 | 0.45 | 0.47 | 0.25 | 0.19 | 0.51 | 0.43 | 0.63 | 0.09 | 0.24 |  | 0.65 | 0.78 | 0.70 | 0.57 | 0.55 | 0.66 | 0.50 | 0.56 | 0.46 | 0.59 | 0.60 | 0.59 | 0.53 |
| 13 | Mpongwe | Zambia | Optimum | 0.37 | 0.41 | 0.38 | 0.45 | 0.33 | 0.22 | 0.42 | 0.62 | 0.64 | 0.10 | 0.31 | 0.79 |  | 0.60 | 0.55 | 0.53 | 0.62 | 0.72 | 0.46 | 0.61 | 0.51 | 0.49 | 0.55 | 0.57 | 0.51 |
| 14 | Lusaka | Zambia | Optimum | 0.36 | 0.47 | 0.40 | 0.55 | 0.29 | 0.15 | 0.60 | 0.52 | 0.75 | 0.24 | 0.37 | 0.96 | 0.73 |  | 0.75 | 0.64 | 0.68 | 0.72 | 0.66 | 0.87 | 0.71 | 0.62 | 0.56 | 0.59 | 0.63 |
| 15 | Gwebi | Zimbabwe | Optimum | 0.55 | 0.46 | 0.41 | 0.41 | 0.37 | 0.18 | 0.51 | 0.58 | 0.78 | 0.11 | 0.28 | 0.98 | 0.76 | 1.00 |  | 0.62 | 0.68 | 0.59 | 0.65 | 0.78 | 0.84 | 0.95 | 0.90 | 0.77 | 0.52 |
| 16 | Rattray-Arnold Research Station | Zimbabwe | Optimum | 0.68 | 0.63 | 0.44 | 0.48 | 0.37 | 0.24 | 0.43 | 0.35 | 0.72 | 0.20 | 0.22 | 0.75 | 0.69 | 0.84 | 0.93 |  | 0.53 | 0.62 | 0.74 | 0.82 | 0.48 | 0.73 | 0.83 | 0.80 | 0.52 |
| 17 | Bako | Ethiopia | Optimum | 0.57 | 0.66 | 0.65 | 0.73 | 0.33 | 0.24 | 0.51 | 0.93 | 0.71 | 0.33 | 0.39 | 0.83 | 0.92 | 1.00 | 1.00 | 0.85 |  | 0.68 | 0.64 | 0.79 | 0.66 | 0.89 | 0.85 | 0.90 | 0.61 |
| 18 | Kiboko | Kenya | Optimum | 0.46 | 0.43 | 0.55 | 0.64 | 0.63 | 0.38 | 0.71 | 0.72 | 0.68 | 0.49 | 0.43 | 0.83 | 0.88 | 0.90 | 0.83 | 0.83 | 1.00 |  | 0.53 | 0.70 | 0.46 | 0.81 | 0.73 | 0.76 | 0.90 |
| 19 | Chiredzi | Zimbabwe | Random Stress | 0.52 | 0.62 | 0.58 | 0.48 | 0.34 | 0.20 | 0.52 | 0.22 | 0.72 | 0.33 | 0.43 | 0.35 | 0.32 | 0.46 | 0.40 | 0.49 | 0.37 | 0.37 |  | 0.40 | 0.38 | 0.34 | 0.47 | 0.32 | 0.44 |
| 20 | Kadoma | Zimbabwe | Random Stress | 0.44 | 0.54 | 0.38 | 0.54 | 0.34 | 0.32 | 0.52 | 0.31 | 0.70 | 0.20 | 0.34 | 0.75 | 0.46 | 0.65 | 0.51 | 0.57 | 0.48 | 0.52 | 0.62 |  | 0.41 | 0.54 | 0.42 | 0.49 | 0.64 |
| 21 | Gweru | Zimbabwe | Random Stress | 0.33 | 0.54 | 0.46 | 0.49 | 0.35 | 0.21 | 0.45 | 0.45 | 0.67 | 0.14 | 0.30 | 0.68 | 0.35 | 0.48 | 0.50 | 0.31 | 0.36 | 0.31 | 0.65 | 0.66 |  | 0.44 | 0.34 | 0.42 | 0.38 |
| 22 | Golden Valley | Zimbabwe | Random Stress | 0.42 | 0.47 | 0.42 | 0.43 | 0.27 | 0.22 | 0.42 | 0.47 | 0.71 | 0.40 | 0.41 | 0.79 | 0.65 | 0.83 | 0.63 | 0.51 | 0.54 | 0.60 | 0.53 | 0.78 | 0.70 |  | 0.42 | 0.56 | 0.62 |
| 23 | Mount Makulu | Zambia | Random Stress | 0.44 | 0.36 | 0.39 | 0.46 | 0.36 | 0.22 | 0.43 | 0.20 | 0.64 | 0.12 | 0.03 | 0.90 | 0.81 | 0.84 | 0.53 | 0.52 | 0.46 | 0.49 | 0.81 | 0.69 | 0.61 | 0.69 |  | 0.47 | 0.47 |
| 24 | Chitedze | Malawi | Random Stress | 0.38 | 0.48 | 0.43 | 0.47 | 0.36 | 0.39 | 0.37 | 0.32 | 0.82 | 0.27 | 0.40 | 0.81 | 0.78 | 0.81 | 0.49 | 0.55 | 0.53 | 0.54 | 0.51 | 0.74 | 0.69 | 0.83 | 0.78 |  | 0.59 |
| 25 | Melkassa | Ethiopia | Random Stress | 0.72 | 0.77 | 0.79 | 1.00 | 0.72 | 0.31 | 0.56 | 0.42 | 0.85 | 0.41 | 0.42 | 0.76 | 0.72 | 0.89 | 0.85 | 0.79 | 1.00 | 0.63 | 0.72 | 0.99 | 0.65 | 0.95 | 0.80 | 0.93 |  |
