# Supplementary figures and images for: Genetic gains in early maturing maize hybrids developed by the International Maize and Wheat Improvement Center in Southern Africa during 2000–2018
Source: Front Plant Sci. 2024 Jan 16;14:1321308. doi: 10.3389/fpls.2023.1321308 (PMC10825029; doi:10.3389/fpls.2023.1321308)

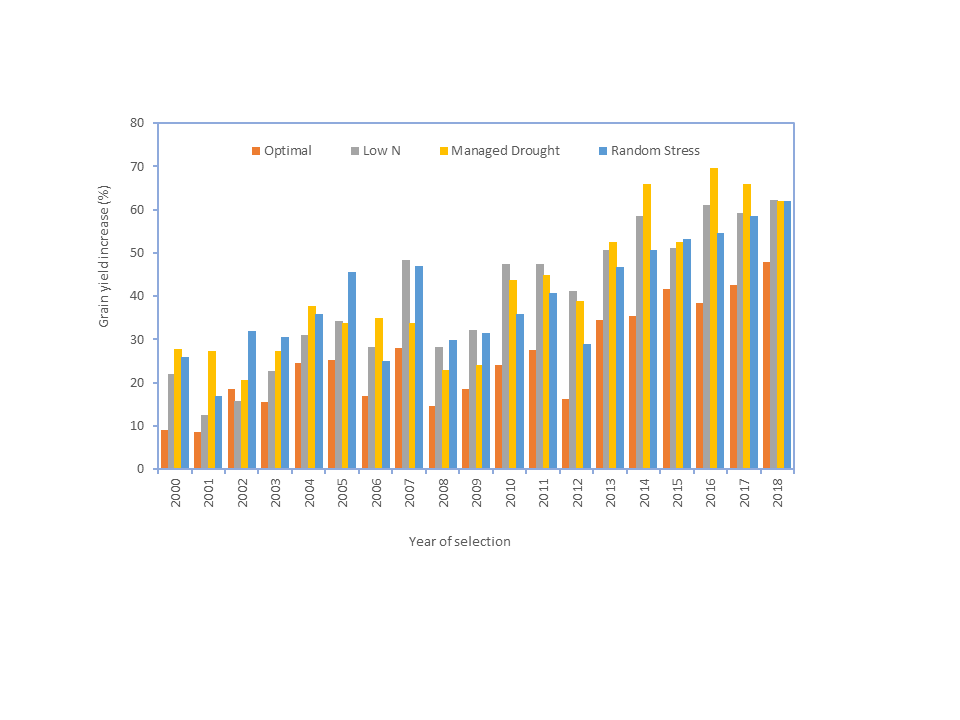

Supplement: Supplementary Figure 1 — Percent grain yield (t ha-1) advantages of era hybrids over the mean of benchmark commercial checks under optimal, low N, managed drought and random stress environments in the era trials conducted in 2018 and 2019. [file Image_1.tif]

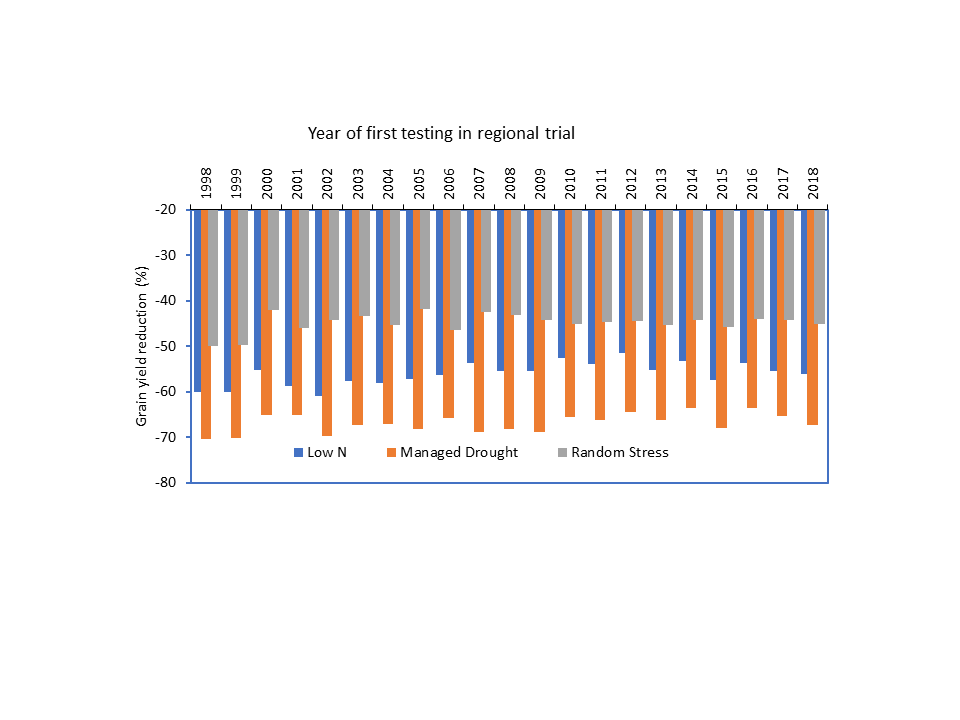

Supplement: Supplementary Figure 2 — Relative grain yield reduction (%) under stress environments as compared to optimal management. [file Image_2.tif]
